# Health shocks and health behavior: a long-term perspective

**DOI:** 10.1007/s10198-024-01747-2

**Published:** 2025-03-26

**Authors:** Christian Bünnings, Irina Simankova, Harald Tauchmann

**Affiliations:** 1 FOM Hochschule, Essen, Germany; 2 Universität Erlangen-Nürnberg, Nuremberg, Germany; 3 CINCH – Forschungszentrum für Gesundheitsökonomik, Essen, Germany; 4 RWI – Leibniz Institute für Wirtschaftsforschung, Essen, Germany; 5 M-CHEP – Munich Center for Health Economics and Policy, Munich, Germany

**Keywords:** Health behavior, Health shock, MCS, PCS, Long-term, Double/debiased machine learning, I12, D12

## Abstract

Several empirical papers suggest that individuals improve health-related behaviors in response to adverse shocks to physical health. However, little evidence exists regarding the questions of (i) how long-lasting these behavioral responses are and (ii) whether individuals respond similarly to mental health shocks. Using individual-level survey data from Germany and combining regression augmented inverse-probability weighting with machine learning prediction algorithms, we compare individuals hit by such shocks to undisturbed individuals up to fifteen years after that shock. The analysis confirms earlier findings that individuals experiencing a sharp deterioration of physical health immediately improve their health-related behaviors in terms of eating more healthily and being less likely to smoke. Contrarily, doing sports is negatively affected. We further find that the immediate response to shocks on mental health is weaker, with the exception of smoking. Tobacco consumption on average becomes more likely after a shock to mental health. We further find that the immediate response to shocks on mental health is weaker, with the exception of smoking behavior, which on average worsens after such kind of a shock. Yet the analysis does not reveal long lasting persistent effects. Significant differences in health behaviors are rarely found more than two years after the shock.

## Introduction

A sudden deterioration of individual health, often referred to as a health shock, is an adverse life event prone to having long-term consequences in various dimensions (e.g. [14, 19, 36, 48]), income, employment, and consumption, for instance.

Besides direct effects on health and economic outcomes, various behavioral responses to such shocks have been discussed in the literature (e.g. [9, 15, 16, 40]). Responses in terms of health-related behaviors are particularly relevant since such behavioral change may be the key to recover from that shock and to avoid further deterioration of health in the future. Somewhat ironically, a healthy lifestyle might already have had reduced the risk of experiencing such shock. In other words, switching to a healthier lifestyle only in response to an adverse shock on health might be regarded as a rather inefficient strategy. However, it is well known that many individuals struggle with adopting a healthy lifestyle though they are well aware of the possible health benefits [24]. Experiencing a sudden worsening of health may have a positive side effect on empowering individuals to achieve such behavioral change. One channel through which this effect may operate is information about health risks gained during the treatment process [12]. Personalized information from the medical professionals during treatment might be more effective in comparison to, for example, prints on the cigarette packages. Another channel might be a change in subjective risk perception and perceived vulnerability to health risks. These arguments can be linked to economic theory. One can, in the spirit of Grossman [21], interpret a health shock—and the subsequent interaction with health care providers—as a source of new health-related information, that makes the individual re-optimize and choose a healthier lifestyle. Margolis [32] —although not explicitly referring to Grossman [21]—brings this argument even closer the Grossman’s model by focusing on education as a mediator. That is, according to this line of argument, better educated individuals are in a better position to process the health information that comes with a health shock and will react more strongly to adverse health events than individuals with less education.^1^ Another economic theory related argument can be based on models such as the seminal model of rational addiction [5]. In the spirit of such model, individuals who have developed unhealthy habits can be interpreted as trapped in a stable steady state from which they cannot escape, even with considerable effort. Such effort may make them improve their health behavior temporarily, yet they will eventually return to their unhealthy habit if the effort slackens. Only a drastic event—a health shock for instance—that destroys much ‘habit capital’ may put the individual on a trajectory that ultimately leads into a different and more healthy equilibrium.^2^

Bad health habits such as poor diet, low level of physical activity, and smoking are the leading causes of preventable death. Complimented by alcohol consumption, these behavioral choices accounted for 4 in 10 deaths in Germany in 2017 [34]. Robert Koch Institute reports that in 2017, the share of German population older than 15 years that smoked was 22.4%; around 16% of adults were obese [28]. Roughly 40% of the German population between 14 and 69 years old do sports less than once a week [46].

Several existing studies address the question of how adverse health shocks affect lifestyles. Most of this research is concerned with smoking behavior. Examples are Bünnings [8]; Clark and Etilé [12, 13]; Marsaudon and Rochaix [33]; Wang et al. [50], which analyze data from Britain, Switzerland, China, and France, respectively. Clark and Etilé [12] and Clark and Etilé [13] find that deterioration of health is associated with reduced future tobacco consumption and an increase in the probability of smoking cessation among smokers. Bünnings [8] establishes similar results for adverse health events provided that they do not concern mental health. This also applies to the analysis of Wang et al. [50]. Although failing to establish effects of health shocks on spousal smoking, which was initially in the focus of their research, they still find such effects on the own smoking behavior.

Using data from the Health and Retirement Study, Smith et al. [41] and Khwaja et al. [25] focus on smoking patterns in the US population of advanced age. Smith et al. [41] address a different—yet related—research question than the earlier mentioned contributions. Rather than analyzing effects on smoking behavior, they study the effect of health shocks on longevity expectation, and compare these effects between smokers, former smokers and never smokers. They find that smokers adjust their subjective life expectancy more strongly in response to a health shock than the two groups of non-smokers do. This can be regarded as evidence that personalized learning about the risks of an unhealthy lifestyle through experiencing an unpleasant health condition may lead to behavioral changes. This idea is also supported by Khwaja et al. [25]. They find that survival expectations respond to own health shocks but not to shocks experienced by others and establish a similar pattern of responses with respect to the decision to quit smoking.

Keenan [23] and Sundmacher [44] do not confine their analyses to effects on smoking behavior, but investigate the effect of adverse health events also on obesity, an outcome closely related to health behavior. Keenan [23] studies the effect of new diagnosis [“stroke, cancer, lung disease (e.g., chronic obstructive pulmonary disease), heart disease (e.g., angina, congestive heart failure, myocardial infarction, or other heart condition), ${\left[ \ldots \right] }$ diabetesmellitus”, [23], p. 238] on weight (except the former two) and smoking (all listed diagnoses) among adults of advanced age using Health and Retirement Study data from 1992 to 2000. According to her findings, individuals with a specific new diagnosis are more likely to quit smoking and to lose weight. Using data from the German SOEP and conceptualizing health shocks as deterioration in self-assessed health, Sundmacher [44] finds that experiencing a health shock has at least a short-term effect on smoking cessation. However, she does not find effects on weight loss.

Temporary changes in health behavior may generate only little health benefits. Knowledge about the persistence of the effect is hence as important as its existence. Short-term effects might be motivated by temporary restrictions, financial or physical ones for instance, rather than a deeper understanding of the risks and re-evaluating personal choices. Unlike the majority of analyses discussed above, Marsaudon and Rochaix [33] do not confine their analysis to short-run effects but also find a significant long-term effect of the health shocks on cigarette consumption. Individuals who experienced a shock smoked after the health deteriorating event on average two cigarettes per week less in comparison to those who did not experience one. Although the time span of the research is substantial (from 1989 to 2014), the sample studied is rather restrictive including only individuals working for the French electricity board.

We contribute to the existing research in four different dimensions. (i) While most of the research focuses only on smoking behavior [8, 12, 13, 25, 33, 41, 50], we study a set of habits such as smoking, dietary preferences, and physical activity. (ii) Since we use data from 2001 to 2019, we have the opportunity to analyze both short- and long-term effects of the shocks. (iii) Much of the existing research [12, 25, 33, 50] is concerned mainly with physical health shock. Following Bünnings [8], we address differential effects of shocks to physical and mental health. (iv) Finally, we contribute to the literature on the effects of health shocks in terms of the empirical methods used. As discussed, health is at least partly determined by behavior and is hence an endogenous variable. Also sudden changes in health cannot be regarded as purely exogenous. Since purely exogenous sources of health variation are hardly identifiable,^3^ most applications rely on conditioning on observables to tackle endogeneity bias. The choice of the conditioning variables is however frequently ad hoc. In contrast, we use machine learning methods, namely, double/debiased machine learning, to condition on covariates in a data driven way. Double/debiased machine learning is designed to estimate treatment effects by employing doubly robust approaches, such as augmented inverse probability weighting. In this framework, nuisance parameters-such as propensity scores and potential outcomes-are estimated using machine learning algorithms, with cross-fitting applied to improve the robustness of these predictions [11].

The paper is structured as follows: in section 2 we describe the data used for the analysis. Then in Sect. 3, the empirical strategy is outlined. Next we present the estimation results and provide the output of a robustness check and heterogeneous effects estimation in Sect. 4. In Sect. 5 we discuss the results and limitations, and finally conclude.

## Data

### Data source

For the empirical analysis, we use the German Socio-Economic Panel (SOEP) v36 [31]. It is a longitudinal survey of the population in Germany that was first conducted in 1984 in West Germany and then was complemented repeatedly with refreshment and special sub-samples in order to maintain the representative nature of the survey. The data are collected at individual and household levels. The SOEP contains information on a wide range of questions regarding socio-economic status, health and lifestyle among many others [47]. In our analysis the individual serves as a unit of observation. We use the SOEP waves from 2001 to 2019.^4^ We do not apply any sample selection criteria as we aim to focus on general population.

The panel structure of the SOEP is key to our research design. Since we are interested not only in the instantaneous effects of health shocks but also in their long-term effects, we need to follow an individual for several years. More specifically, we focus on health shocks that occur at a specific point in time and link them to health behaviors observed in several waves of the SOEP from 2004 to 2019. That is, separate analyses are carried out with different health behaviors observed at different points in time serving as outcome variables.

**Fig. 1:**
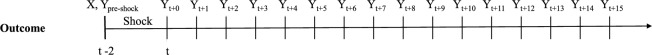
General description of the timing structure

Understanding timing structure in this study is crucial. Therefore, we first introduce the general design of the research and how the estimation data are organized. Subsequently, we describe how the key variables, that is, outcomes and health shocks, and in addition the covariates are measured. Figure 1 illustrates the basic concept that applies to any type of outcome-shock combination. We separately estimate the effect of a shock on an outcome $Y_{t+s}$ at different time points after the shock occurred between $t-2$ and *t*, with period *t* being fixed at a specific year depending on the outcome. That is, *s* runs from 0 (contemporaneous effect) to *S* (most long-term effect). We choose to fix *t* at a specific year instead of allowing shocks to occur at any time. This approach is designed to replicate an experimental setting in which potential future events that happen after the treatment is administered or not are excluded from consideration. Thus, the key aspects of the design are: (i) the shock happens between period $t-2$ and *t*, the fixed period *t* serves hence as reference; (ii) to estimate the effect of a shock on the outcome we use $Y_{t+s}$ as dependent variable in a homogeneous estimation procedure. This allows us shedding light on how possible effects evolve over time.^5^ We start with an initial estimation sample for the first observed period after the occurrence of the shock (sample for $t+0$). Then, due to the attrition from the SOEP, the estimation sample diminishes in size for increasing time-lags between the shock and measurement of the outcome.^6^

### Dependent variables

As outcome variables we consider three health-related lifestyles, namely, (i) not smoking, (ii) keeping a health-conscious diet, and (iii) doing sports at least once a week. More precisely, these variables are defined as follows: (i) an individual does not currently smoke cigarettes, or a pipe, or cigars with values 1 indicating ‘yes’, and 0 indicating ‘no’.^7^ Smoking is hence not confined to cigarettes consumption but covers a broader set of tobacco products. The question about the individual smoking status is asked in the survey every even year from 2002. (ii) An individual follows health-conscious diet with 1 standing for ‘very much’ or ‘much’, and 0 indicating ‘little’ or ‘not at all’. The corresponding question is asked in the SOEP every even year starting from 2004 until 2014. (iii) An individual does sports regularly, with values 1 indicating ‘at least once a week’ and 0 indicating ‘less frequent’. For this outcome we use information from every odd survey year starting from 2001 in order to maintain the biannual structure of the data.^8^

**Fig. 2:**
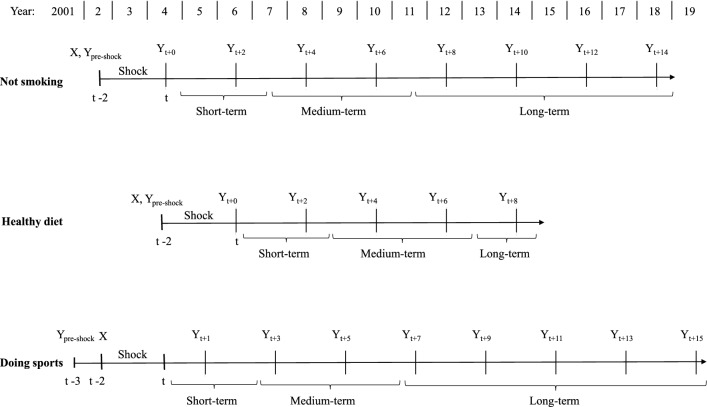
Description of the timing structure

**Fig. 3:**
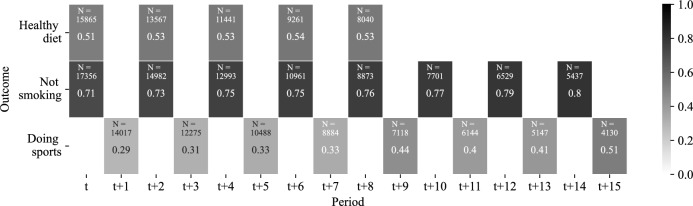
Distribution of the outcome variables by years from shock. Note: the scale corresponds to the share of individuals with ’yes’ answer. Thus, the table reads as follows, e.g. upper left cell: There are 15,865 individuals in the estimation sample of *diet*_*t*+0_ with 51% keeping health-conscious diet

As pointed out, information on health behaviors is not available annually but—depending on the specific behavior—in different waves. This renders the structure of the working data somewhat complex with respect to the time span considered between the health shock and the measurement of the respective outcome. Figure 2 visualizes the structure of the data in terms of the different points in time at which the outcomes are observed. Figure 3 complements Fig. 2 providing information on the respective sample sizes and on the distribution of the outcomes for different values of *s*. The biggest sample size for the variable *not smoking* is 17,356 and the smallest 5,437; for *healthy diet* the corresponding figures are: 15,865 and 8,040; for *doing sports* 14,017 and 4,130, respectively.

Figure 3 presents descriptive statistics for the outcome variables at different points in time.^9^ To provide a brief overview: the share of non smokers ranges between 71% and 80% across samples. The share of those who keep a healthy diet ranges from 51% to 53%, we consider it to be quite stable as the time horizon increases. For the share of those doing sports regularly we observe that it increases with time and ranges between 29% and 51%.^10^

### Health shocks

We define health shocks based on the Physical Component Summary Scale and Mental Component Summary Scale (MCS/PCS). These well established health measures are based on the SF-12 questionnaire, which since 2002 is a part of the SOEP every second year. They are widely regarded as compact and reliable indicators of individual health [1]. Using MCS and PCS as health measures also has much appeal from a conceptual perspective. Both measures are derived from answers to a wide range of health-related survey questions by the means of a factor analysis [1]. MCS and PCS, hence, allude to the idea of health as a latent variable that cannot directly be observed but still underlies any specific measure of health. In consequence, defining health shocks in terms of changes in MCS and PCS allows for not confining the analysis to very specific health events but considering also changes in latent general health. Though MCS and PCS allow for measuring health more generally than considering very specific health outcomes would do, they maintain the idea that two different major domains of health exist, physical health and mental health. This concept of two major latent health domains is regularly warranted by the factor analysis used for deriving the two summary scales. The values of MCS and PCS range from 0–100, with a higher score indicating the better mental and physical health, respectively. MCS and PCS are normalized to have a mean of 50 and standard deviation of 10. We define shocks to mental and physical health in a similar fashion as Bünnings et al. [9] and Li et al. [30]. That is, the shock indicators take the value of 1 if a reduction by more than 25% in the respective component score is observed, relative to its level two years earlier, that is, $\mathbbm {1}\left( \frac{\text {MCS}_t - \text {MCS}_{t-2}}{\text {MCS}_{t-2}} < -0.25\right)$ and $\mathbbm {1}\left( \frac{\text {PCS}_t - \text {PCS}_{t-2}}{\text {PCS}_{t-2}} < -0.25\right)$. We check for the robustness of the results to choice of the threshold value; see Sect. 4.2.

Though the levels PCS and MCS are orthogonal by construction in the norm population, this does not imply uncorrelatedness of the two types of health shocks. Yet, in the estimation sample this correlation is rather small and negative, and does not significantly differ from zero; see Table 2 for details. This finding backs the idea of thinking about mental and physical health as different health domains.

Considering dietary preferences, we look at the effect of shocks that occurred between 2004 and 2006. For *not smoking* and *doing sports*, we evaluate the effect of a shock that happened between 2002 and 2004; see Fig. 2.^11^ The years 2004 and 2006, respectively, thus serve as reference. We hence compare the respective outcome between individuals hit and individuals not hit by a shock *s* years after the reference year. The analysis is therefore unconditional on further health events that may or may not occur at a later time. This mimics a straightforward experimental design in which one would also not condition on post-treatment events. However, when interpreting estimated effects, especially long-term ones, it is important to bear in mind that the control group does not exclusively consist of people who have never experienced a shock. We distinguish between shocks to mental and shocks to physical health and separately analyze their effects. These two kinds of health shocks may very differently affect health-related behaviors. With respect to smoking, Bünnings [8] provides an example for such pattern of heterogeneous effects. He finds that while a shock to physical health increases the probability of smoking cessation, a shock to mental health has the opposite effect.

**Fig. 4:**
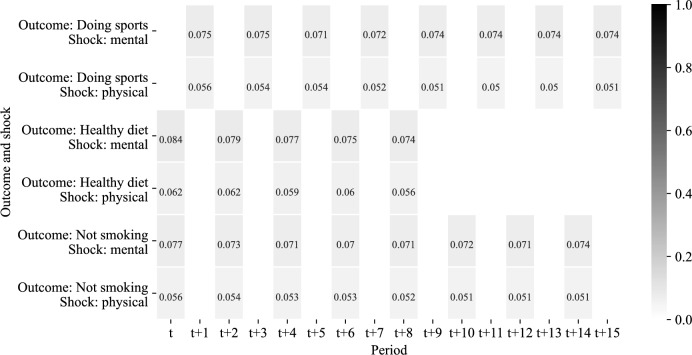
Distribution of the treatment variables by years from shock and type of shock. Note: the scale corresponds to the share of individuals that experienced a health shock. Thus, the table reads as follows, e.g. upper left cell: Among the estimation sample for analyzing the instantaneous effect of shock to mental health on doing sports, 7.5% of individuals experienced a mental health shock. Only shocks happening between the reference period t and the previous period *t* − 2, i.e. for not smoking and doing sports – 2002 and 2004, for healthy diet 2004 and 2006, are considered

The proportion of individuals experiencing a health shock is rather small, around 5%−8%, with shocks to mental health being more frequent by roughly two percentage points (Fig. 4). Figure 10, found in the appendix, provides the distributions of the change in component scores between periods *t* and $t-2$.^12^ We descriptively also look at repeated health shocks, i.e. whether the individuals who experience a shock tend to experience further ones in the future. Yet, we find little evidence for such pattern being common. Only few individuals are observed to be hit by more than two health shocks during the time period 2002 to 2018 (Table 3).

Selective attrition from the sample may hamper the analysis of long-term effects. To address this concern, in Fig. 4 we report the shares of individuals hit by a health shock for all estimation samples. The sample sizes get smaller if longer time horizons are considered, which is explained by panel attrition. Yet, the shares of individuals hit by a health shock in 2004 (2006, respectively) stay almost constant over time. In addition, we look at cell-specific attrition rates – with cells being defined by treatment status and respective outcome – as the share of the sample for analyzing the effects at period $t+0$ (Table 4). We observe that the attrition rates do not differ much across the cells. This descriptive finding argues against health shock driven attrition being a serious issue.

### Covariates

Conditioning on observables is a key to our empirical analysis. The SOEP contains very broad information at the individual and household level from which we extract a rich subset of potential controls.^13^ All information on the covariates is measured prior to the reference year, that is, ‘pre-treatment’. More specifically, the set of covariates consists of 40 variables. Six variables are continuous, namely, MCS, PCS, body mass index, age, size of the household, and education. The rest of the variables are binary dummies, indicating German nationality, being from East Germany, being female, marital status (married, single), having children, employment status (unemployed, retired), occupation (blue collar worker, white collar worker, civil servant, self-employed), being disabled, self-assessed health (good, satisfactory, bad), overnight hospital stay [based on the previous year], doctor visits [based on the previous quarter], satisfaction with several dimensions of life, namely, health, living situation, income, leisure time, and life in general (three categories each), and income (four categories). Table 5 provides descriptive statistics for the covariates, on the basis of estimation sample for analyzing smoking in $t+0$.^14^ In addition, we condition on the pre-shock level of the respective outcome variable $Y_{t-2}$.

Table 1 provides descriptive statistics of the outcome variables measured pre-treatment ($Y_{t-2}$). Regarding the outcomes *not smoking* and* doing sports*, the share of those having a good habit in sub-sample of individuals who experienced a shock is consistently significantly lower in comparison to the sub-sample of those who did not experience one. The share of respondents keeping a health conscious diet is higher among those who experienced a shock though the difference is significant only considering physical shock. This pattern suggests that the occurrence of health shocks is not independent of past health behavior and that conditioning on pre-shock health behaviors is necessary.

**Table 1:** Descriptive statistics of the outcome variables measured pre-treatment ($Y_{t-2}$)

Outcome	Shock	Overall		With shock		W/o shock		p-value
		$N_{t+0}$	$Y_{t-2} = 1$	$N_{t+0}$	$Y_{t-2} = 1$	$N_{t+0}$	$Y_{t-2} = 1$	
Not smoking	Physical	17,356	0.70	977	0.67	16,379	0.70	0.024
Not smoking	Mental	17,356	0.70	1,337	0.66	16,019	0.70	0.005
Healthy diet	Physical	15,865	0.51	990	0.54	14,875	0.51	0.039
Healthy diet	Mental	15,865	0.51	1,333	0.53	14,532	0.51	0.134
Doing sports	Physical	14,017	0.26	789	0.19	13,228	0.27	0.000
Doing sports	Mental	14,017	0.26	1,057	0.24	12,960	0.27	0.034

Note: The measurements are based on the estimation sample of the corresponding outcome in $t+0$ period. *N* is the number of individuals in the (sub)sample; $Y_{t-2} = 1$ refers to having a good habit in a pre-shock period. The table reads as follows, e.g. there are 17,356 individuals in the estimation sample of $not smoking_{t+0}$, 70% of whom did not smoke in period $t-2$; out of 977 respondents that experienced a physical health shock between 2002 and 2004, 67% did not smoke in period $t-2$; out of 16,379 who did not experience a physical health shock – 70% did not smoke. The p-value corresponds to the Pearson’s Chi-squared test of equal means

**Fig. 5:**
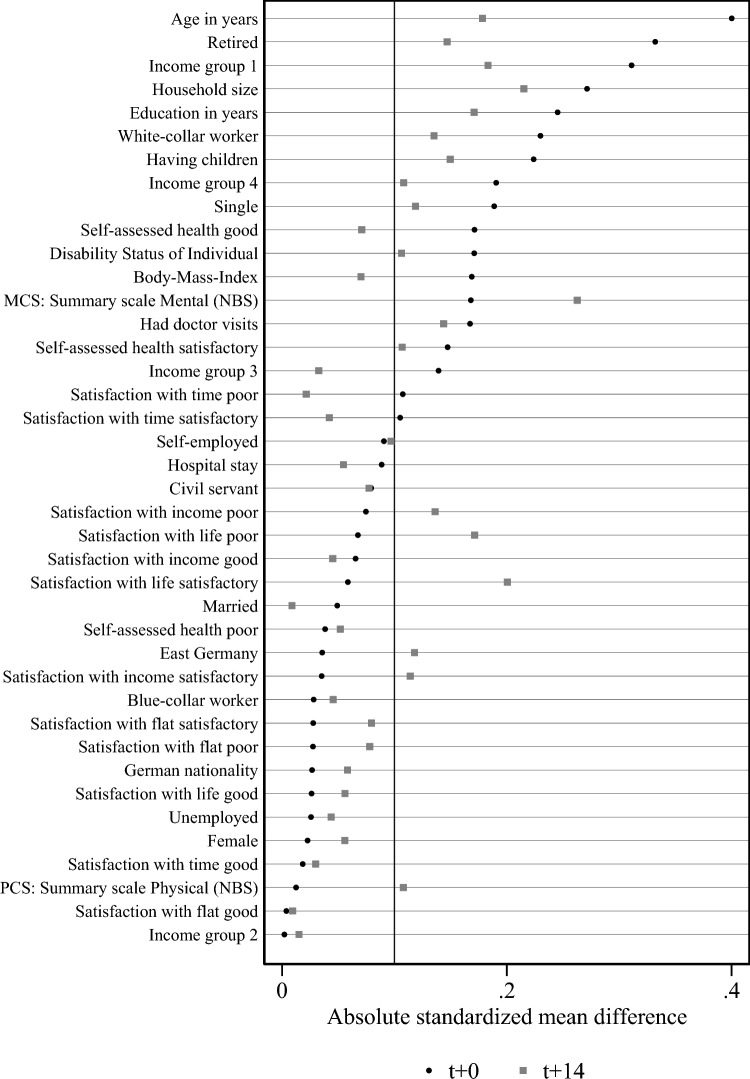
Absolute standardized mean differences. Note: the results are based on the samples for analyzing the effect of a shock to physical health on smoking behavior

Beyond describing the covariates for the full estimation sample, we check how balanced they are between the group of those hit and those not hit by a shock. For this purpose, we use the absolute standardized mean differences (ASMD)^15^. According to this measure, a deviation of more than 0.1 indicates a lack of balance, which may point to non-random assignment to the two groups [3]. Figure 5 depicts the absolute standardized mean differences in the samples for analyzing smoking behavior in periods $t+0$ and $t+14$ (maximum years from shock). While the deviation is smaller than 0.1 for the majority of covariates, some deviate substantially between the two groups. Treatment and control groups differ the most in terms of age, retirement status, being very poor based on equalized household income. Between $t+0$ and $t+14$, the group deviations become smaller for most variables that exceed the threshold of 0.1 in $t+0$ (upper rows of the graph) yet gets bigger for variables that showed a particularly small deviation in the initial estimation sample (lower rows of the graph). Finding a lack of balance between treatment and control suggests using a balancing procedure, e.g. inverse probability weighting, prior to evaluation of effect.

## Methods

Though health shocks are typically perceived as exogenous events that are not under the control of the individuals who experience them, they are nonetheless not purely randomly assigned. As discussed in section 2, numerous socioeconomic characteristics—such as age, occupation, household composition, years of education, et cetera—are associated with the likelihood of experiencing a sudden deterioration of health. Non-random treatment assignment challenges the estimation of health shock effects, and may result in erroneously attributing the impact of confounding factors to the treatment effect of interest. Education, for instance, is (negatively)^16^ correlated with experiencing adverse health events, compare Fig. 5. Yet, at the same time, ample empirical evidence suggests that individuals with more years of education exhibit, at least with respect to some behaviors, more healthy live styles [7, 29, 35]. We address this issue through conditioning on observables.^17^ More specifically, we combine doubly robust treatment effect estimation, namely, augmented inverse-probability weighting [20, 27, 37], with machine learning techniques, double/debiased machine learning (DDML) [10, 11] in particular. The former only requires modeling the role confounders play correctly either for the process that determines the outcome or for the process of treatment assignment. The latter allows for considering a large number of possible confounding variables, without specifying an overly rich model and without relying on pretest estimators, which render statistical inference invalid. Our empirical analysis aims at estimating the average treatment effects (ATE), that is, we estimate the expected effects health shocks exert on an individual randomly sampled from the population.^18^$\tau ^{\text {AIPW}}_{\text {ATE}}$ denotes the doubly robust estimator of that quantity, namely augmented inverse-probability weighting (AIPW).

### Augmented inverse-probability weighting

Augmented inverse probability weighting (AIPW; [37]) combines two approaches to conditioning on observables: (i) Estimating outcome models for treated and untreated units, predicting the potential outcomes under treatment and under no treatment conditional on a set of confounders *X*—denoted *g*(1, *X*) and *g*(0, *X*)—and subsequently contrasting the estimated potential outcomes. (ii) Modeling treatment assignment as a function of confounders to estimate propensity scores ${\pi (X)= {{\,\textrm{P}\,}}(T=1 | X)}$ [38, 39], that is, the probability of receiving the treatment *T* conditional on *X*. Weighting the observed outcomes by the inverse probabilities, ${1}/{\pi (X)}$ and ${1}/{(1-\pi (X))}$ respectively, hence adjusts the comparison of outcomes for the confounders *X* affecting the treatment status. Under the assumptions of stable unit treatment values (SUTVA), common support, and conditional independence (CIA)^19^ either approach allows for consistent estimation of the treatment effects. Combining two methods of conditioning on observables renders AIPW doubly robust,^20^ which means that consistency is preserved even if the outcome model violates conditional independence, as long as the treatment assignment model satisfies the CIA, and the other way round.

The algebraic representation of the AIPW estimator below (cf. [20]), with *i* indexing observational units and *N* denoting the sample size, depicts how the two approaches to conditioning on observables are combined.

1 $$\begin{aligned} \hat{\tau }^{\text {AIPW}}_{\text {ATE}}&= \frac{1}{N} \sum _{i = 1}^{N} \left[ \left( \frac{T_i \cdot Y_i}{\hat{\pi }(X_i)} - \frac{T_i -\hat{\pi }(X_i)}{\hat{\pi }(X_i)} \hat{g}(1,X_i) \right) - \left( \frac{(1 -T_i) \cdot Y_i}{1 -\hat{\pi }(X_i)} + \frac{T_i -\hat{\pi }(X_i)}{1-\hat{\pi }(X_i)} \hat{g}(0, X_i) \right) \right] \end{aligned}$$

Dropping the terms involving $T_i -\hat{\pi }(X_i)$ from (1) just yields the pure inverse probability weighting estimator (IPW). This implies for the AIPW that if the treatment assignment model does a good job in predicting the actual treatment status $T_i$, and in consequence $T_i -\hat{\pi }(X_i)$ gets small, the outcomes predicted under treatment $\hat{g}(1, X_i)$ and under no treatment $\hat{g}(0, X_i)$, obtained from estimating the outcome models, receive little weight. If, however, the treatment assignment model does a poor job in predicting $T_i$, the outcome model contributes much to the estimated average treatment effect. In fact, for an extremely poor performance in predicting the treatment status, the treatment model would become immaterial. Moreover, the adjustment terms involving $T_i -\hat{\pi }(X_i)$ counterbalance the sensitivity of the pure IPW to estimated propensity scores taking values close to zero or unity ([20], p. 40). After all, if the outcome model predicts the observed outcome very well and $Y_i -\hat{g}(1,X_i)$ and $Y_i -\hat{g}(0,X_i)$ approach zero for treated and untreated units, respectively, then only the outcome model matters for $\hat{\tau }^{\text {AIPW}}_{\text {ATE}}$. This becomes obvious from rewriting the right-hand-side of (1) as $\frac{1}{N} \sum _{i = 1}^{N} \left[ {T_i \cdot \left( Y_i -\hat{g}(1,X_i) \right) }/{\hat{\pi }(X_i)} - { (1-T_i)\cdot \left( Y_i -\hat{g}(0,X_i)\right) }/{(1-\hat{\pi }(X_i))} +\hat{g}(1,X_i) - \hat{g}(0, X_i)\right]$.

### Double/debiased machine learning

Though AIPW has the double robustness property, consistent estimation still requires that the CIA holds at least for the treatment assignment model or the outcome model. If the available data are very rich, one can condition on a huge number of potential confounders and confounder interactions. One may, hence, argue that this brings one at least close to conditional unconfoundedness. However, conditioning excessively results in a problem of high dimensionality, which may render model estimation very imprecise if not technically intractable [18]. By selecting a manageable set of variables to enter *X* just by economic intuition, one may miss important confounders. If the conditioning variables are selected on basis of preparatory regression results, one ends up with a pretest estimator that may result in severely misleading inference [49]. Machine learning methods provide a way out of this dilemma by efficiently using the information in a possibly huge set of conditioning variables in a data driven way.

Subsequently, we follow a recent framework suggested by Chernozhukov et al. [11]. This double/debiased machine learning approach uses some machine learning algorithm for estimating the nuisance parameters *g*(1, *X*), *g*(0, *X*), and $\pi (X)$. In order to avoid over-fitting, the procedure relies on sample splitting, i.e. different sub-samples are used for estimating (training data) and actually predicting (test data) the nuisance parameters. Cross-fitting, that is, switching between the roles of training and test data, improves the efficiency of the procedure by allowing to obtain predictions for the entire sample. In a nutshell, the idea behind the algorithm is the following ([4, 42], p. 374):

(i) The sample is randomly partitioned into *K* splits. $N_k$ denotes observations that fall into *k*th split, with $k = 1,..., K$. $N_{-k}$ denotes the rest of the sample.
(ii) The nuisance parameters are estimated on $N_{-k}$. For this step of the estimation procedure various machine learning algorithms can be used. Then, these parameters are used to make predictions $\hat{g}(1,X_{ik})$, $\hat{g}(0, X_{ik})$, and $\hat{\pi }(X_{ik})$ for the $N_k$. This procedure is repeated *K* times, such that predictions for all the observations of sample are made.
(iii) The ATE is calculated using (1).
(iv) In order to avoid sampling error due to partitioning, the above steps are repeated *R* times, using new resamples $r = 1,..., R$, that is, new partitions of the sample into splits. Resampling and averaging limits the impact that unfortunate random splits of the sample may have on the result. Finally, the median of the *R* results for $\hat{\tau }^{\text {AIPW}}_{\text {ATE},r}$ is calculated and reported as $\hat{\tau }^{\text {AIPW}}_{\text {ATE}}$.

In step (ii) we use random forest to estimate *g*(1, *X*) and *g*(0, *X*) and $\pi (X)$.^21^ To prevent over-fitting, we tune the maximum depth of the decision tree by testing different values: 3, 4, 6, 8, 10, 12, 14, 16, 18, and 20. We use three-fold cross-validation to evaluate each option, and then chose the best model. Tuning is done for each split in every resample, *R*, which is set to 15. In each tree, a subset of all possible confounders (features) is considered, with its size equaling the square root of the total number of features. In each forest, 500 trees are estimated and the results are averaged. In a robustness check, LASSO is used as an alternative machine-learning approach for the estimation of nuisance parameters.^22^

## Results

### Main Results

**Fig. 6:**
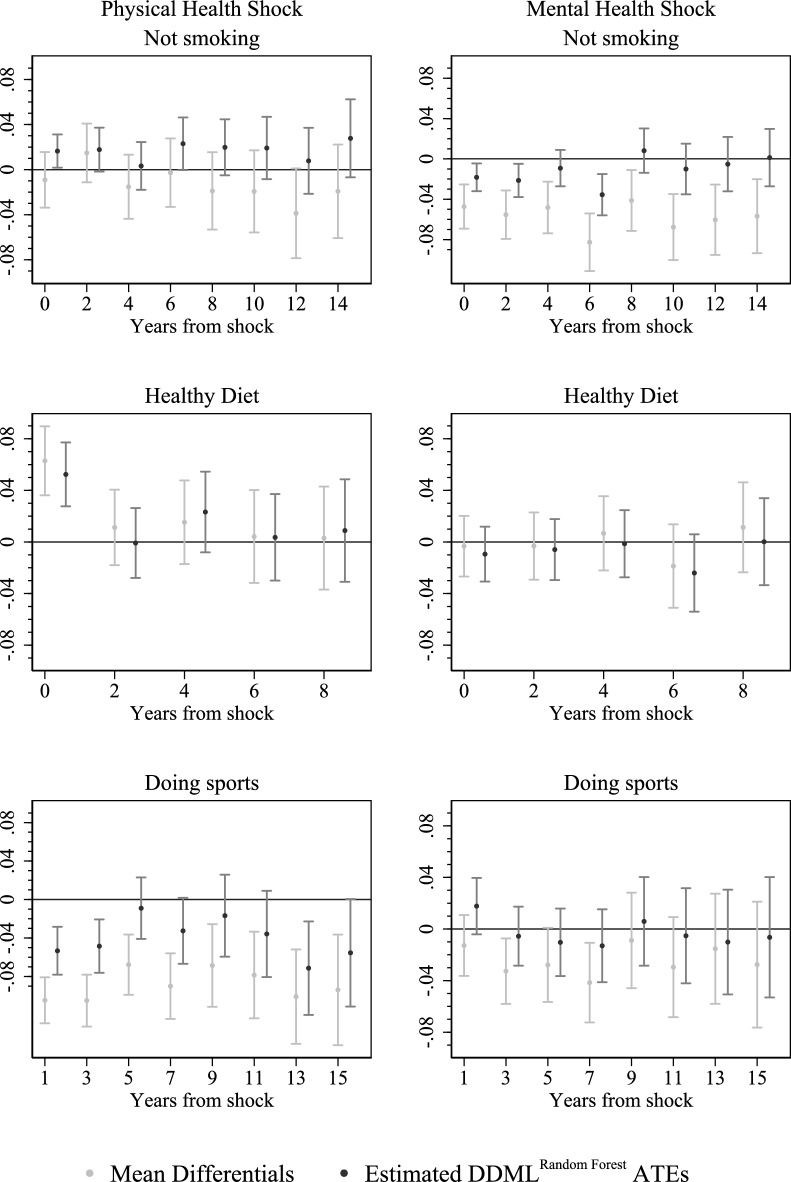
Estimated $\text {DDML}^{\text {RF}}$ ATEs vs. Mean Differentials. Note: 90% confidence intervals are represented by vertical lines

In this section, we firstly outline the results produced using the above mentioned methodology. Later on, robustness checks and treatment effects heterogeneity are discussed. As a start, we describe the results from doubly robust machine learning estimation using random forest ($\text {DDML}^{\text {RF}}$) and compare them to the descriptive benchmark, i.e. the group-mean differentials in the three outcome variables at different points in time. To present the large number of estimated effects (three outcomes, two shocks, several different time lags) in an accessible way, we rely primarily on a graphical representation; see Fig. 6. The effect for each lifestyle-shock combination is shown on a separate graph, where the estimated effects are plotted against years from the shock. The reported point estimates are accompanied by estimated 90% confidence intervals.

To begin with, Fig. 6 indicates that the $\text {DDML}^{\text {RF}}$ estimates do not differ much from their descriptive counterparts^23^. This, in particular, applies to the outcome *healthy diet*. There the $\text {DDML}^{\text {RF}}$ point estimates almost coincide with the respective raw group-mean differentials for either type of shock and also for any considered time-lag. For the outcome *doing sports*, deviations in the results are more pronounced. There $\text {DDML}^{\text {RF}}$ yields point estimates that are in absolute terms smaller than what the simple descriptive approach suggests. This finding appears plausible since double robust estimation aims on eliminating selection bias that most likely is away from zero.^24^ Nevertheless, the patterns of how the estimates evolve with increasing time from the shock is very similar and the estimated confidence intervals heavily overlap (except for very short-term analysis of a shock to physical health). That is, also for the outcome *doing sports*, $\text {DDML}^{\text {RF}}$ estimation only slightly changes the pattern of results a naive descriptive analysis yields. With respect to the outcome *not smoking* the picture is more heterogeneous. Regarding the effect of a shock to mental health, $\text {DDML}^{\text {RF}}$ estimation puts a persistent adverse effect suggested by the naive analysis into question, albeit confirming adverse short-term effects. Regarding a shock to physical health, $\text {DDML}^{\text {RF}}$ and the naive comparison of group means frequently yield point estimates of opposite sign. Yet, in quantitative terms this discrepancy still appears to be moderate when compared to sampling error quantified by the confidence intervals. All in all, the finding that results from $\text {DDML}^{\text {RF}}$ estimation do not differ fundamentally from simple group mean comparisons may be interpreted such that health shocks are random to a major extent and less endogenous than one may hypothesize. We nevertheless base the subsequent detailed discussion on the result from $\text {DDML}^{\text {RF}}$ estimation, since this approach is more robust an involves much weaker implicit assumptions.

With respect to smoking behavior, the immediate effect of a physical health shock on the probability of not smoking is roughly 2 percentage points (pp) and is statistically significant at the 10% level. Compared to a baseline not smoking rate of 70% this appears to be a rather moderate effect, in quantitative terms. For longer time-lags between the shock and the measurement of the outcome, the point estimates are still positive yet the effects lose statistical significance. In contrast, the estimation results suggest that immediately after a shock to mental health the probability of not smoking decreases by roughly 2pp. This effect appears—with the exception of a lag-length of 6 years—to become smaller for longer time-horizons and finally fluctuates around zero when approaching the end of the observation period. The results hence suggest that smoking behavior responds to sudden deterioration of health only in the short run. The direction of this response differs depending on whether mental or physical health is affected. Our results do not contradict previous studies that find that a general health shock is positively associated with smoking cessation [12, 13]. Moreover, the effect we estimate for a mental shock is qualitatively in line with the findings of Bünnings [8]. Yet, most importantly, our results cast doubts on these short-term responses developing into long-term behavioral change.

Looking at the outcome* healthy diet*, we see that physical shocks on average exert an immediate positive effect of roughly 5pp, which is statistically significant. That is, individuals tend to keep a more healthy diet right after the adverse event. Nevertheless, this effect, as well, appears to be moderate given that anyway one in two individuals keeps a healthy diet at baseline. However, already after two years the effect drops substantially and then fluctuates around zero for longer time-horizons. For the mental shock, there is no significant effect on self-reported healthy diet, even not immediately after the adverse event. Our result of adverse health events having very little, in particular, no sustained effect on nutrition related behavior corresponds with Sundmacher [44] who—also using the SOEP—finds no effect of health events on weight loss.^25^

Finally, we examine effects on the outcome *doing sports*. Figure 6 indicates a negative effect of the worsening of physical health, which is statistically significant for several distances from time of the health shock (namely, 1, 3, and 13 years from the shock). In quantitative terms it ranges from 1pp to 7pp, which is relatively strong compared to a rate of 26% by which routinely exercising is observed at baseline. Physical exercise is hence the only considered outcome that appears to be almost persistently affected. Evidently, various different channels may contribute to this effect. One channel, by which the persistent negative effect may well be explained, are physical restrictions in consequence of the adverse health event, which restrain individuals from exercising. The effect of mental health shock is, in terms of the point estimates, very small and mostly negative. Unlike for shocks to physical health, the estimates are throughout statistically insignificant. The data, hence, provide no evidence for a response of engaging in physical exercise to a worsening of mental health.

All in all, we do not observe much in terms of behavioral change in response to experiencing a sudden deterioration of health. While we find some short-term effects, our findings do not support the hypothesis that an adverse health shock acts as a catalyst that enables individuals bringing themselves to persistently adopting more healthy lifestyles.

### Robustness checks

In this section we provide only few robustness checks as a lot of decisions regarding the model specification are done implicitly in a data driven fashion by the $\text {DDML}^{\text {RF}}$ algorithm. Nonetheless, to assess the robustness of results discussed in the previous section we: (i) check for how sensitive the results are to the choice of the machine-learning algorithm; (ii) examine the robustness to alternatively defined health shocks; (iii) address the concern that conditioning on observables from the pre-treatment period is not sufficient for dealing with heterogeneous pre-treatment trends by (a) estimating placebo effects and (b) conditioning on the history of observables.

**Machine-learning algorithm **We re-estimate the ATEs using LASSO (cf. [45]) for covariates selection instead of Random Forest.^26^ While the general DDML framework is not changed by using a different machine-learning algorithm, LASSO is still the more parametric choice of the estimation procedure. The results from LASSO hardly differ from those Random Forrest estimation yields; see Fig. 15. As the only prominent exception, for the longest time-lag between shock and measurement of the outcome LASSO yields a very wide estimated confidence interval for the effect of a physical shock on keeping a health-conscious diet. This, however, is of no importance to the economic interpretation.

**Health shock definition **We proceed with addressing the robustness of treatment variable definition. Since a health shock is not unambiguously defined we test how robust our results to alternative definitions, in particular with respect to the threshold of 25% that renders a worsening in health a shock. For this purpose we choose alternative threshold values, ranging from 1% and 40% with the step of 1, and re-estimate the effects on behaviors; see Fig. 12, Fig. 13 and, Fig. 14 in the Appendix. Most importantly, the results are rather robust to moderate adjustments of the definition of a health shock. Not surprisingly, this does not apply considering extreme threshold values. If only extreme changes in PCS and MCS are considered shocks, the results get very unstable, and occasionally rather large point estimates occur. This can be explained by health shocks then becoming very rare events making very few observations drive the results. The estimated effects appear to get smaller if very small threshold values are used and, in consequence, even very minor changes in MCS and PCS are counted as shocks. This can be attributed to such measure probably capturing more noise than genuine health deterioration. All in all this robustness check does not suggest that the key result of little behavioral response is an artifact of choosing the threshold of 25% for defining a shock to physical and mental health.

**Fig. 7:**
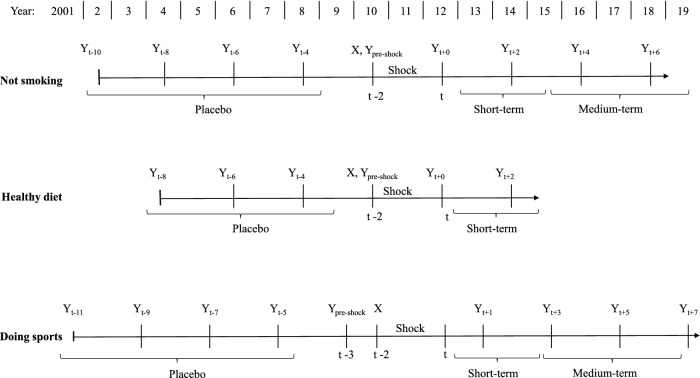
Description of the timing structure of the identification test

**Fig. 8:**
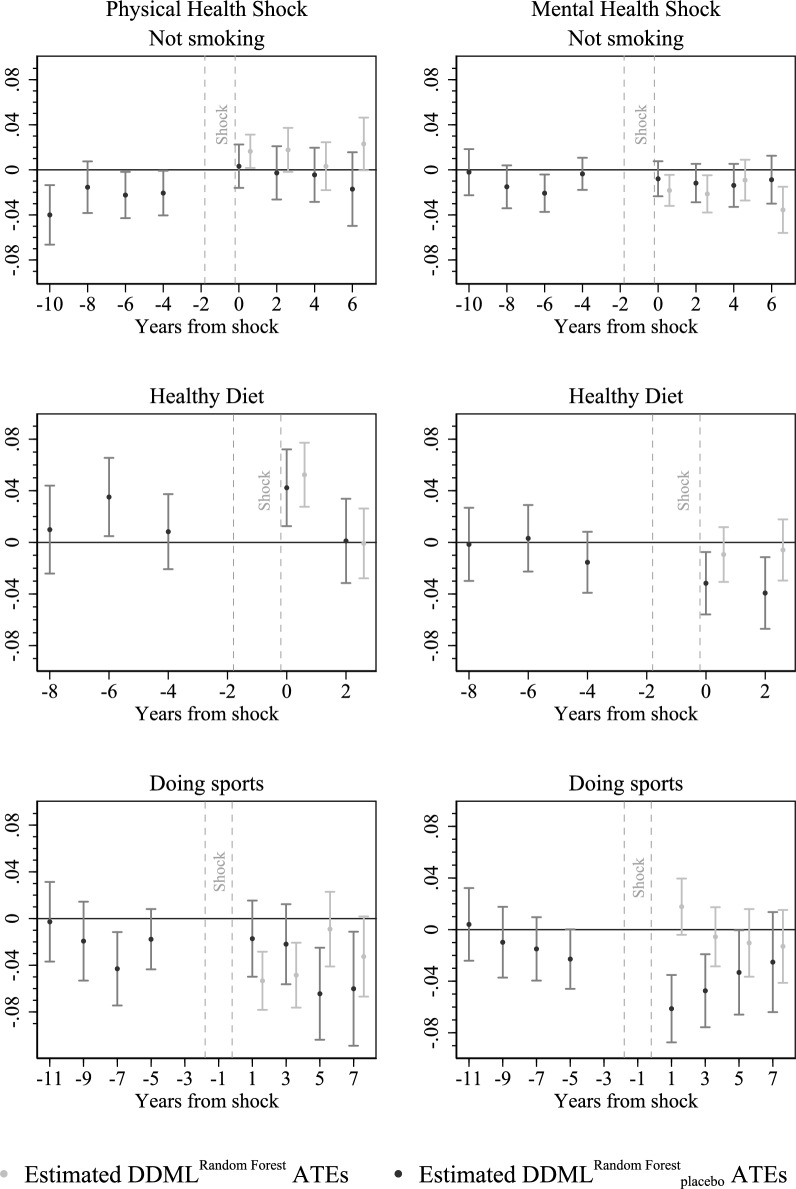
Identification test: Estimated $\text {DDML}^{\text {RF}}$ ATEs vs. Estimated $\text {DDML}^{\text {RF}}_{placebo}$ ATEs. Note: 90% confidence intervals are represented by vertical lines

**Pre-treatment trends **One might argue that the balancing procedure is not sufficient to eliminate possible treatment endogeneity as the groups may differ historically regarding the pre-treatment development of observables, pre-treatment outcomes in particular. In other words, conditioning on only controls measured in $t-2$ might not be enough. In order to evaluate how successful our strategy in terms of different pre-trends issue, we perform identification tests. The idea is to shift the two-years interval in which we measure the shock several periods ahead and to look at the pre-treatment effects expecting them not to deviate from zero significantly. Thus, instead of looking at shocks happening between 2002 and 2004, we look at shocks that occur between 2010 and 2012 (Fig. 7). The results of the identification tests are presented in the Fig. 8.^27^ (Note that gray dots do not indicate simple group mean differences but the corresponding DDML estimates discussed in section 4.1.)

We observe some pre-treatment effects that are significantly different from zeros at 10% level, namely in samples $not \, smoking_{t-10}$ – physical health shock, $not \, smoking_{t-6}$  – physical health shock, $not \, smoking_{t-6}$ – mental health shock, $diet_{t-6}$ – physical health shock, $sports_{t-7}$ – physical health shock. Consequently, we cannot clearly rule out different pre-trends of control and treatment groups. Therefore, we conduct another robustness check where we condition not only on covariates measured in period $t-2$ but in all the other observed pre-shock periods. In order to have reasonable amount of pre- and post-treatment periods, we keep the year when a shock takes place between 2010 and 2012 as it was in the identification tests. Consequently, for the outcome *not smoking* we include covariates that vary over time measured in $t-2$ to $t-10$; for the outcome *healthy diet in *$t-2$ to $t-6$; for the outcome *doing sports* in $t-2$ to $t-10$.

**Fig. 9:**
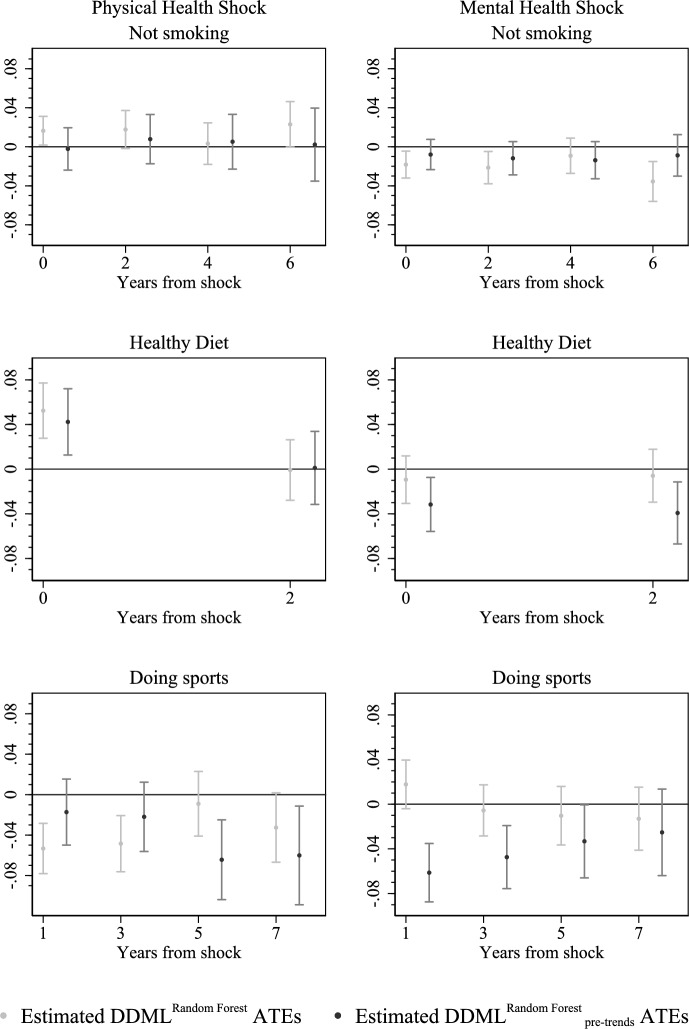
Estimated $\text {DDML}^{\text {RF}}$ ATEs vs Estimated $\text {DDML}^{\text {RF}}_{pre-trend}$ ATEs. Note: 90% confidence intervals are represented by vertical lines; the covariate set for estimating DDML_pre−trend_^RF^ ATEs includes covariates measured in all observed pre-treatment periods

**Fig. 10:**
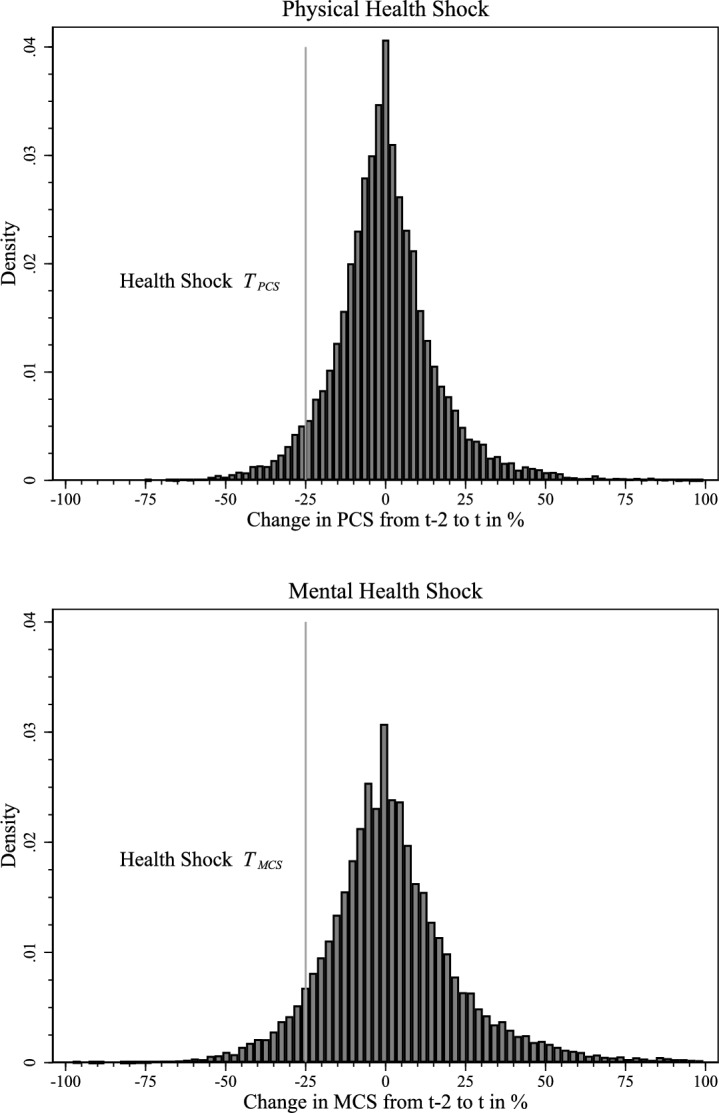
Distribution of change in component scores (smoker sample)

**Fig. 12:**
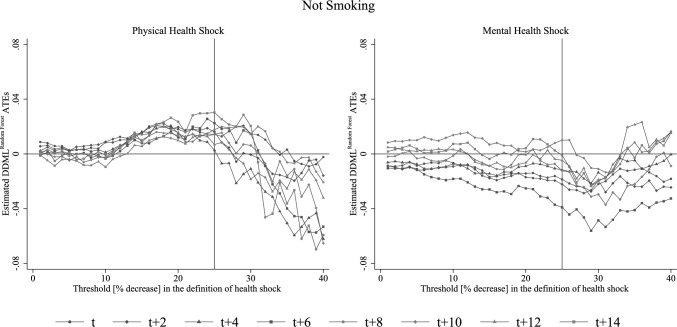
Robustness check: threshold

**Fig. 13:**
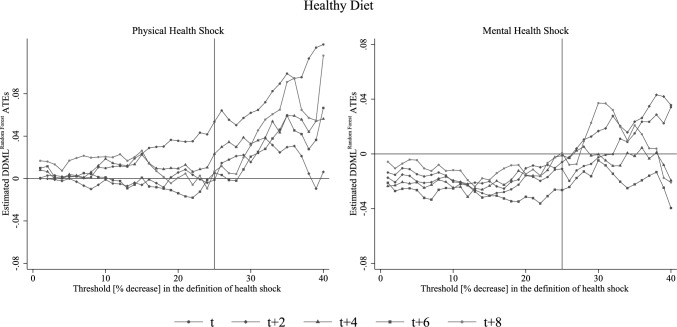
Robustness check: threshold

**Fig. 14:**
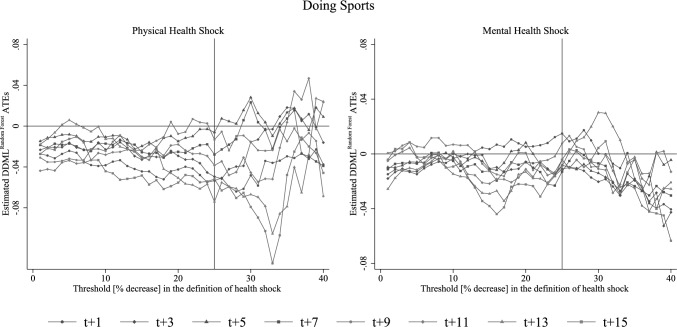
Robustness check: threshold

**Fig. 15:**
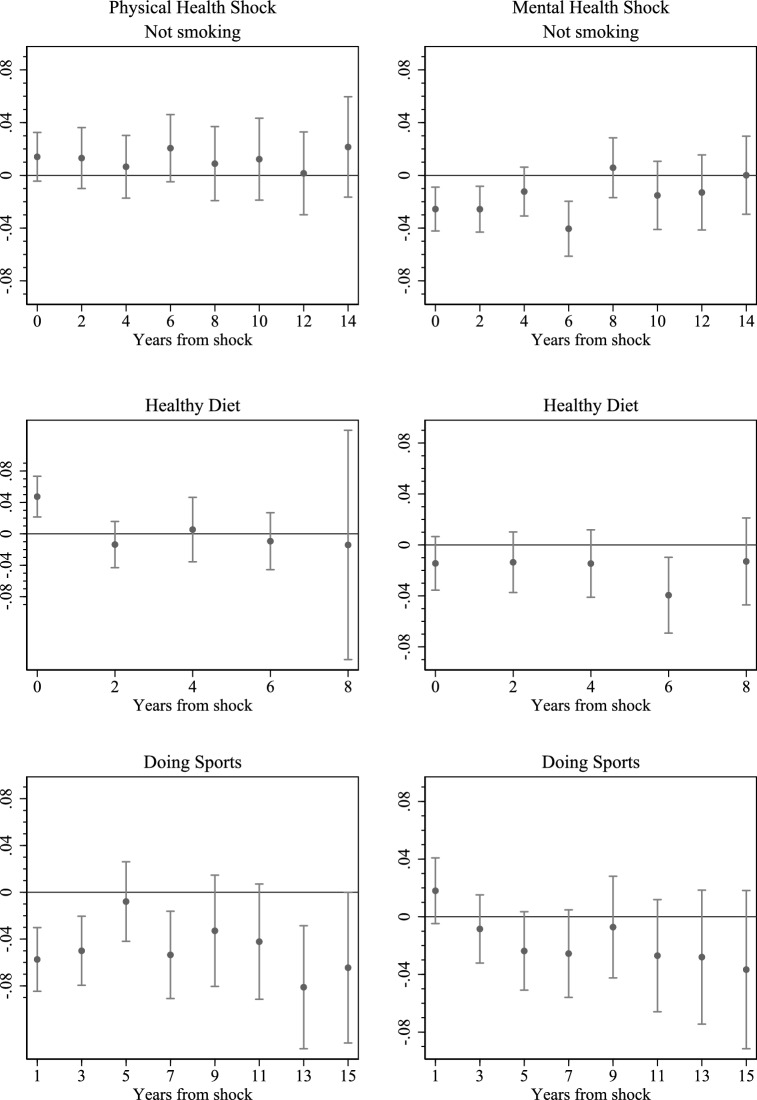
Estimated $\text {DDML}^{\text {LASSO}}$ ATEs. Note: 90% confidence intervals are represented by vertical lines

After conditioning on covariates measured in several pre-treatment periods (Fig. 9), any effect on smoking behavior disappears. Yet, taking the estimated confidence bands into account, the robustness check still yields the results that are not too different in comparison to the main ones. Regarding the outcome *healthy diet*, the effect of a shock to physical health almost coincides with the estimates of preferred specification. Looking at the effects of a shock to mental health, we now observe slight negative effects in the periods $t+0$ and $t+2$ (−3pp). Yet, as above, the estimated confidence intervals overlap heavily which does not suggest that conditioning on more pre-treatment information makes a substantial difference. Finally, the short-term effects of a shock to physical health on doing sports at least once a week turn insignificant, while mid-term effects vice-versa become significant, amounting to roughly −6.5pp and −6pp. Here again, the robustness check yields different results only in terms of the point estimates. With regard to mental health shock, both short-term and mid-term effects turn significant up until $t+5$ period. The effects are diminishing with time, starting with −6pp in period $t+1$ and reaching −2.5pp in period $t+7$. The short term effect on doing sports is indeed the only estimate for which also the confidence bands suggest that our earlier result does not survive the robustness check. All in all, the point estimates proved to be somewhat sensitive to conditioning on the entire history of control variables. Yet the changes in estimates still appear to be rather small compared to the substantial statistical uncertainty they are subject to. The results from our preferred estimation approach, in particular significant short-term effects, have to be interpreted with much caution. However the robustness check confirm that large instantaneous and mid-term effects are not found in the data. Only with respect to the outcome *doing sports* the conclusion is slightly more ambiguous.

**Selective panel attrition **In order to deepen our understanding of whether our results are—at least in part—artifacts of selective panel attrition, we complement the empirical analysis with auxiliary estimations that go beyond the purely descriptive approach discussed in Sect. 2; see also Table 4 in the appendix. Specifically, we treat panel attrition as another outcome that is analyzed in the same way as the behavioral outcomes in the reference estimations. Technically, attrition is defined as not providing information on the respective behavioral outcome and is therefore specific to the three health behaviors considered and also captures temporary non-response. Figure 28, which shows the results of these auxiliary analyses, is therefore parallel to the earlier presentation of the results and provides six sub-figures. The simple comparison of means shows that sample attrition is consistently and statistically significantly higher in the treatment group than in the control group. However, the pattern is not as clear when the analysis is done conditional on the pre-shock covariates using $\text {DDML}^{\text {RF}}$, especially when the confidence intervals are taken into account.^28^ Only for a mental health shock and the sample used to estimate the effects on a healthy diet, does the analysis show a statistically significant and persistently higher attrition rate among those who experienced a shock. In contrast, there are hardly any significant differences in attrition rates when a physical health shock is considered in the samples used to estimate the effects on *not smoking* and on *doing sports*. However, even if we were to give more credence to the purely descriptive evidence, we don’t see this as a serious challenge to our earlier conclusions. Panel attrition and temporary non-response are often related to adverse life events, with death being the most extreme example. Consequently, the group that suffers more from attrition and non-response is more positively selected. That is, the descriptive selection pattern we see in the estimation samples would most likely exaggerate positive behavioral responses to health shocks. However, we nonetheless find almost no such responses, especially in the long run.

### Effect heterogeneity

In order to figure out whether the near absence of significant average effects just hides heterogeneous effects at the subgroup level, we stratify the analysis and compare the estimated effects between subgroups. We begin with exploring drivers of heterogeneity in the effects. In order to figure out what variables it might be, we look at the squared semi-partial correlations ($SPC^2$) of estimated individual-level effects^29^ and covariates. The $SPC^2$ measures the share of independent variation in the individual-level effects that can be attributed to a conditioning variable.^30^ The $SPC^2$ for all lifestyle-shock combinations in every period are presented in Figs. 19, 20,  21. The figures do not reveal a striking pattern of few conditioning variables persistently driving the heterogeneity in the estimated effects. We look closer at some lifestyle-shock combinations at specific time periods for which our preferred estimation procedure yields effects significantly different from zero^31^. (Figs. 22, 23, 24, 25, 26, 27). The most determining variables differ from combination to combination. Yet being unemployed, being single, being satisfied with health and leisure time, being German and being female frequently appear among the variables that contribute most to explaining effect heterogeneity.

**Fig. 16:**
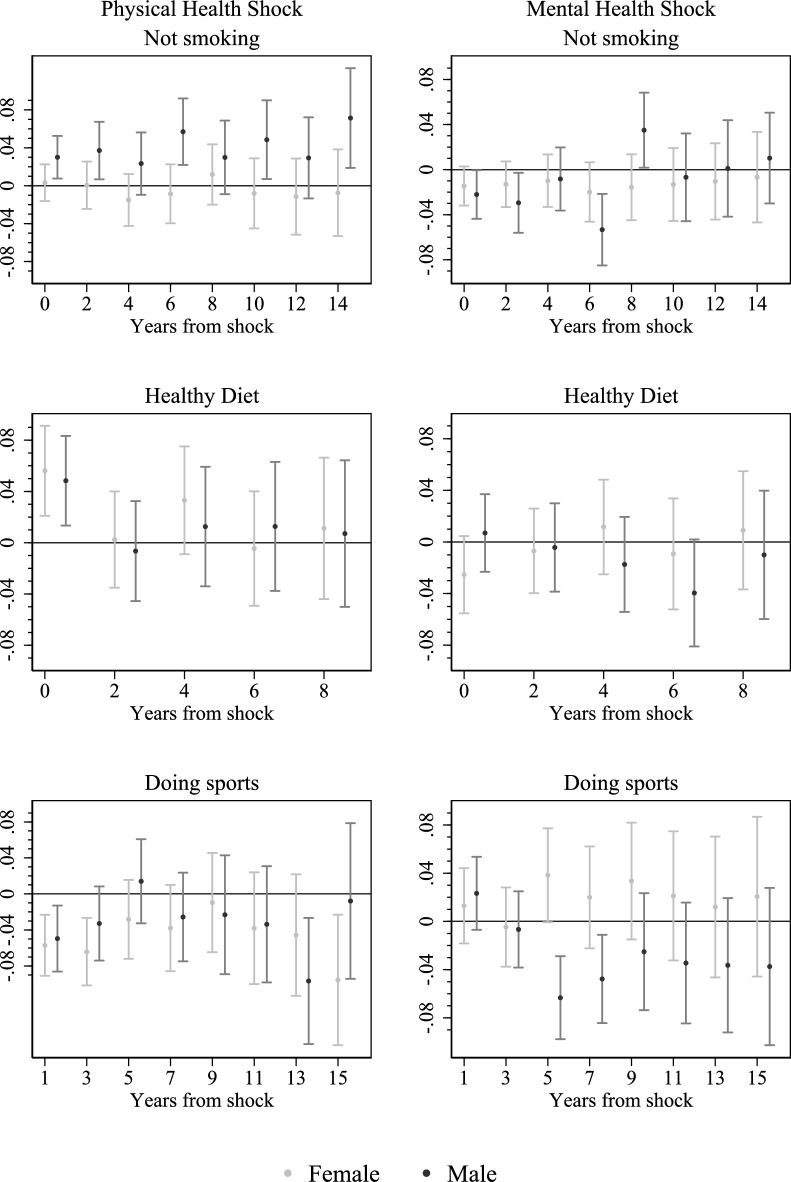
Estimated $\text {DDML}^{\text {RF}}$ ATEs: Female vs. Male. Note: 90% confidence intervals are represented by vertical lines

**Fig. 17:**
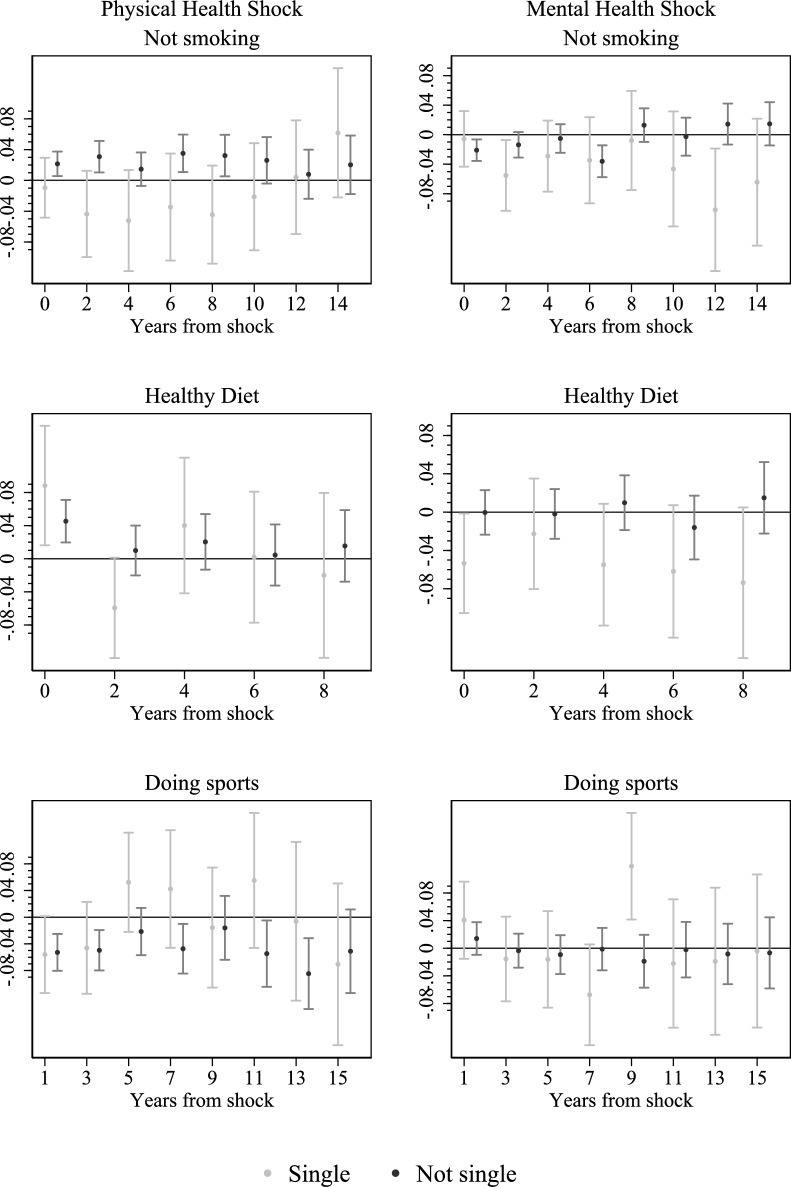
Estimated $\text {DDML}^{\text {RF}}$ ATEs: Single vs. Not Single. Note: 90% confidence intervals are represented by vertical lines

**Fig. 18:**
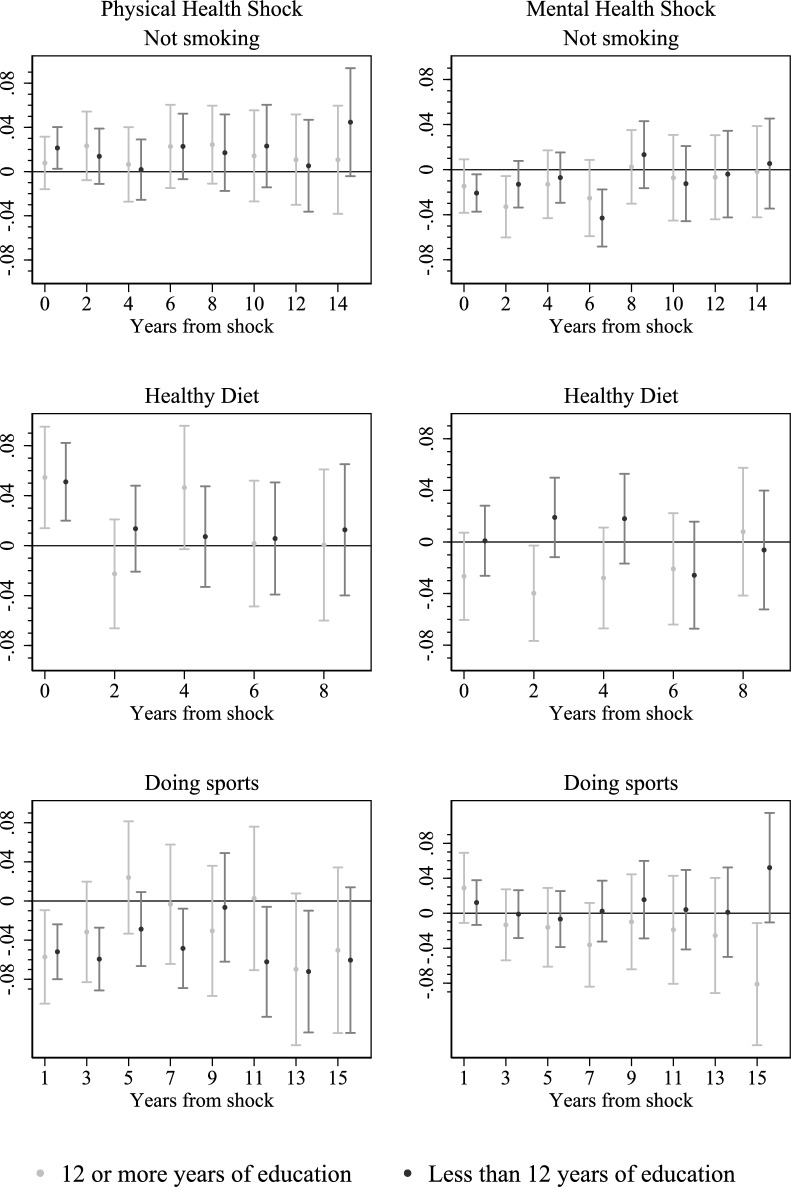
Estimated $\text {DDML}^{\text {RF}}$ ATEs: 12 or more vs. less than 12 years of education. Note: 90% confidence intervals are represented by vertical lines

**Fig. 19:**
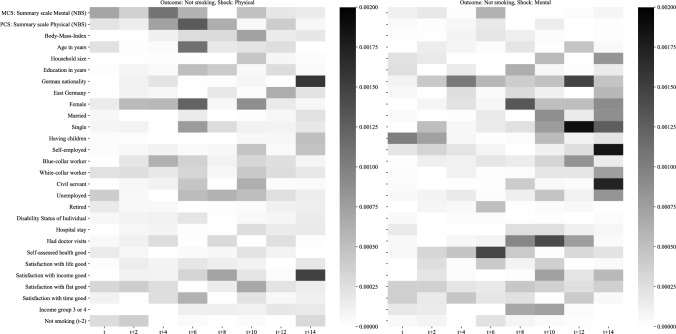
Heterogeneity analysis: squared semi-partial correlation (*not smoking*)

**Fig. 20:**
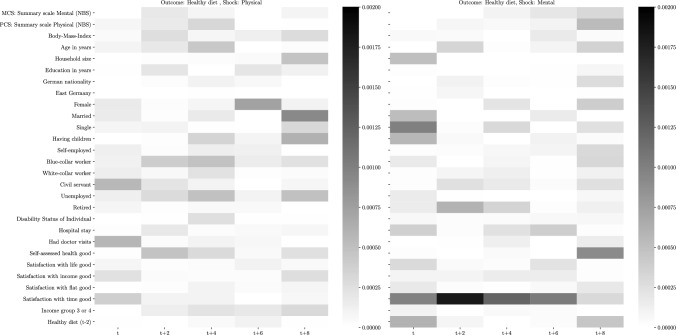
Heterogeneity analysis: squared semi-partial correlation (*healthy diet*)

**Fig. 21:**
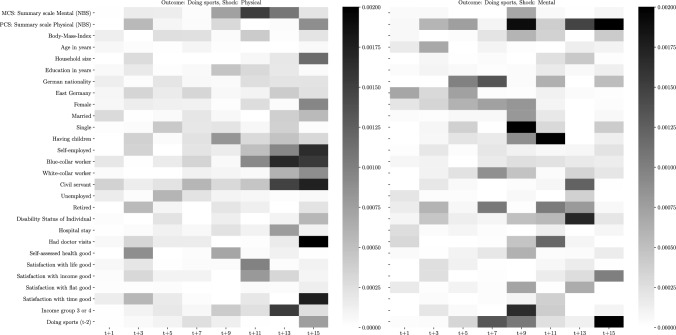
Heterogeneity analysis: squared semi-partial correlation (*doing sports*)

**Fig. 22:**
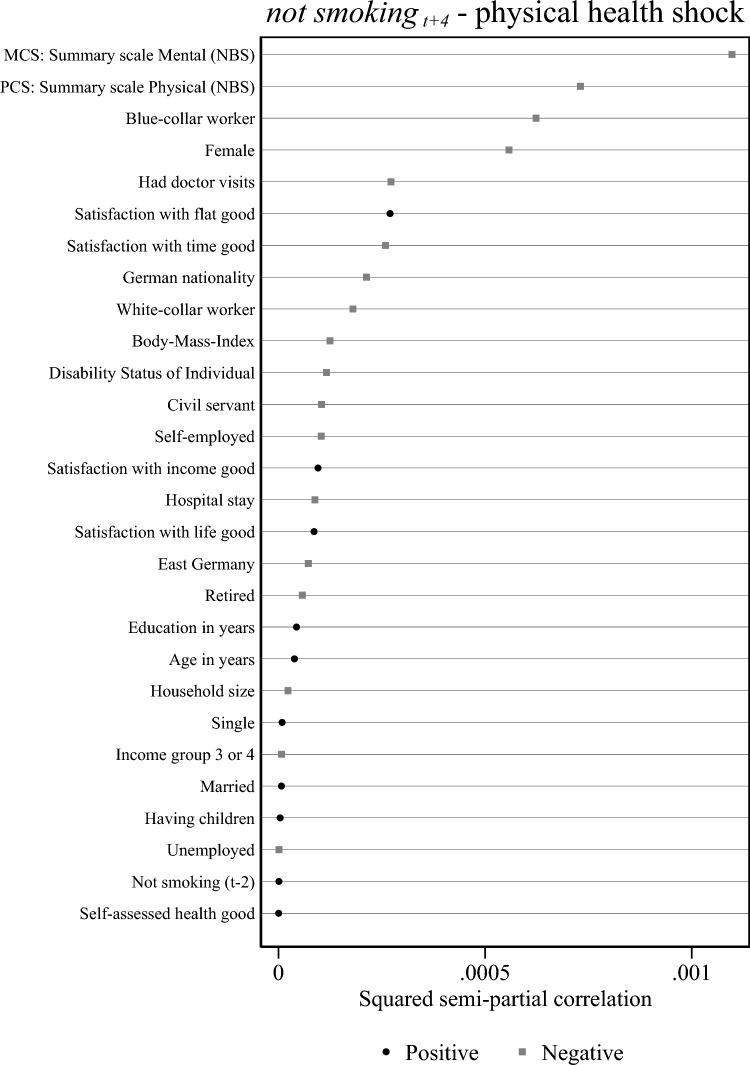
Heterogeneity analysis: Not smoking. Note: while the depicted values represent squared semi-partial correlations, positive and negative distinguishing refers to the sign of semi-partial correlation

**Fig. 23:**
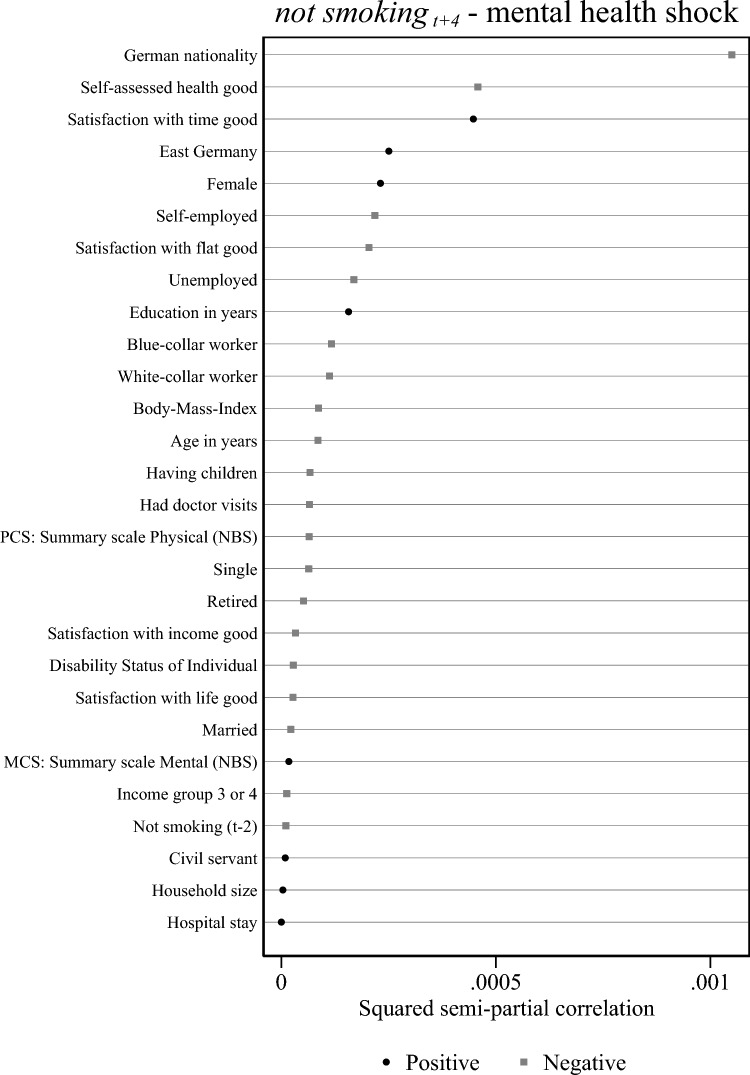
Heterogeneity analysis: Not smoking. Note: while the depicted values represent squared semi-partial correlations, positive and negative distinguishing refers to the sign of semi-partial correlation

**Fig. 24:**
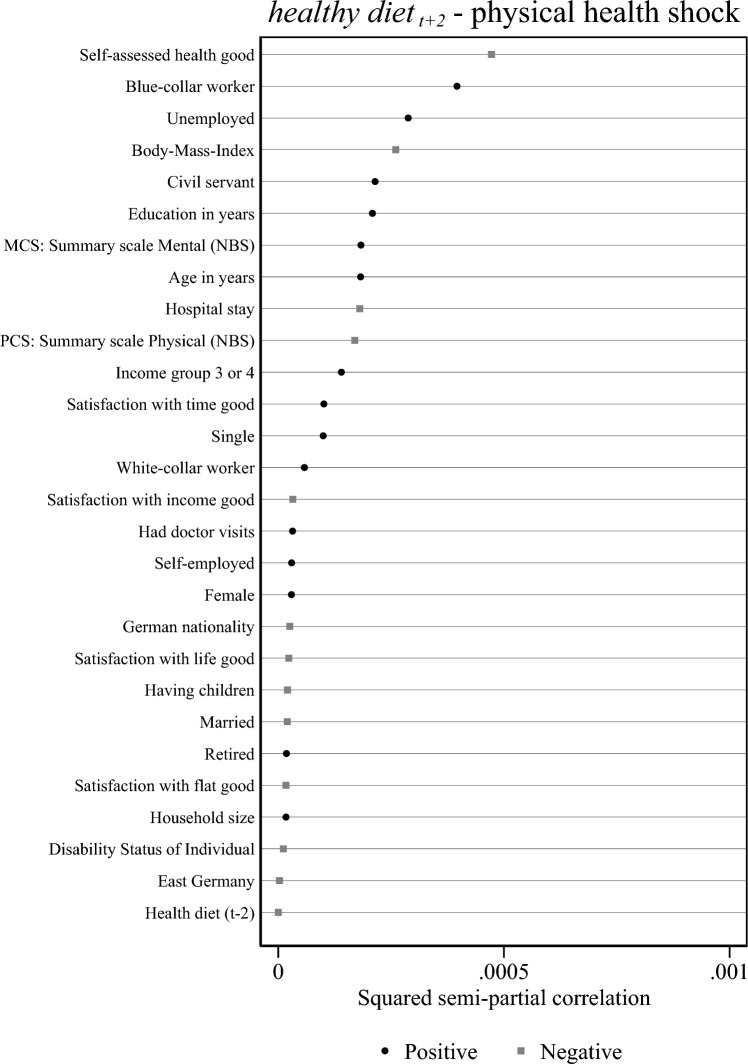
Heterogeneity analysis: Healthy diet. Note: while the depicted values represent squared semi-partial correlations, positive and negative distinguishing refers to the sign of semi-partial correlation

**Fig. 25:**
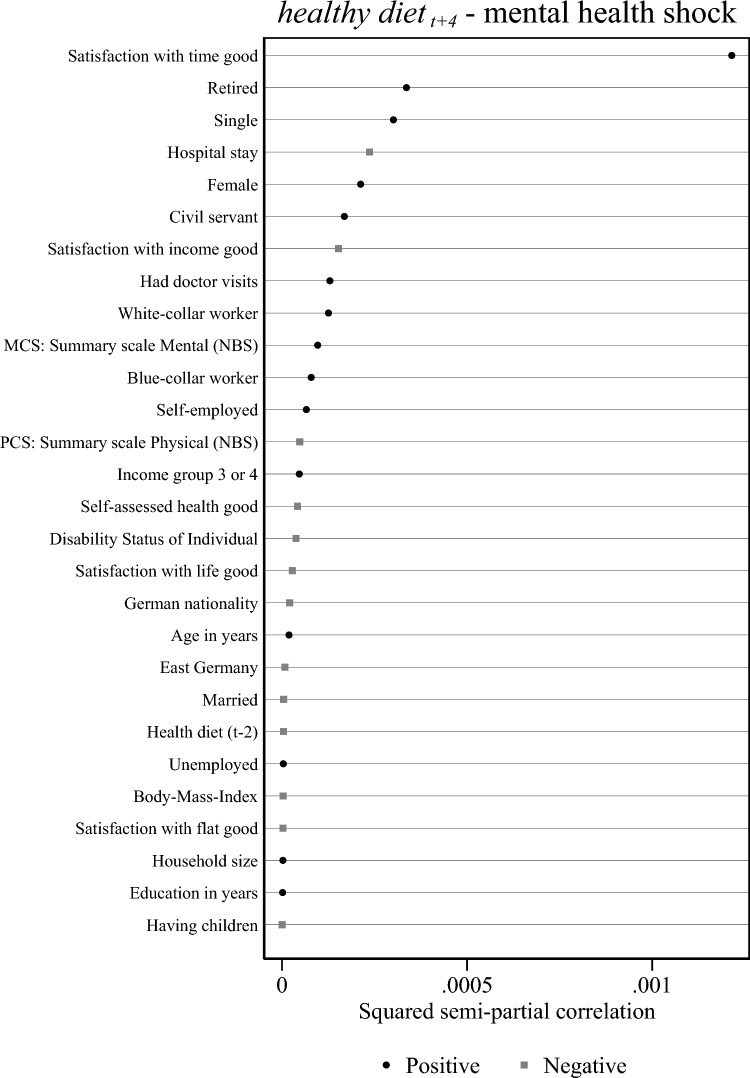
Heterogeneity analysis: Healthy diet. Note: while the depicted values represent squared semi-partial correlations, positive and negative distinguishing refers to the sign of semi-partial correlation

**Fig. 26:**
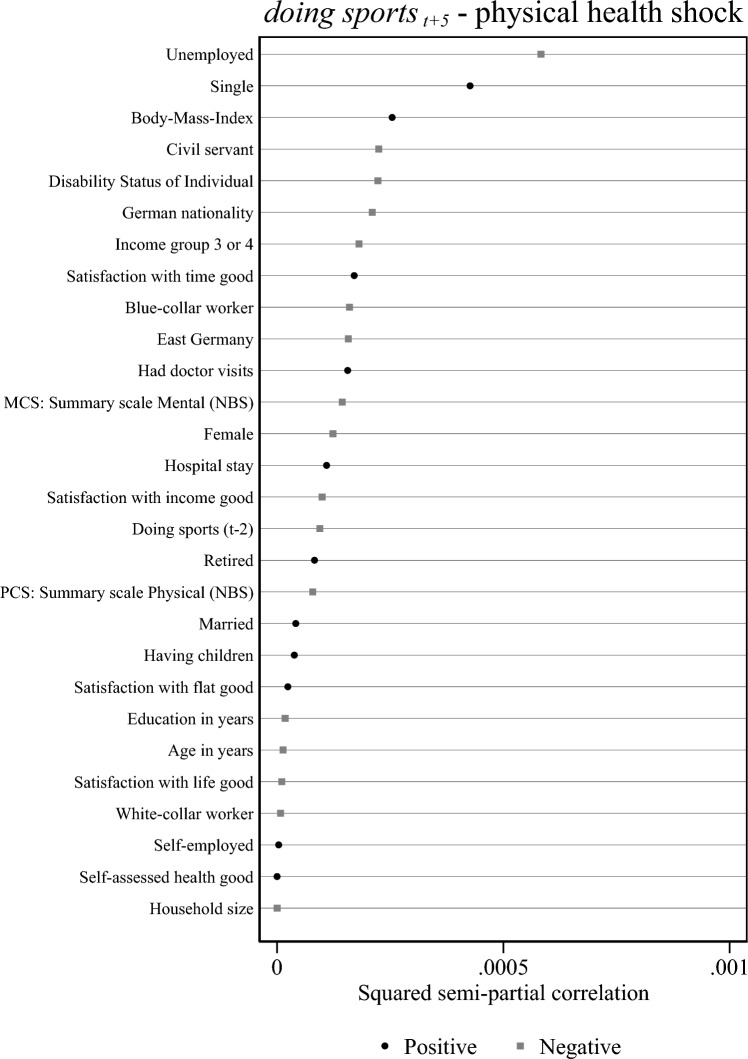
Heterogeneity analysis: Doing sports. Note: while the depicted values represent squared semi-partial correlations, positive and negative distinguishing refers to the sign of semi-partial correlation

**Fig. 27:**
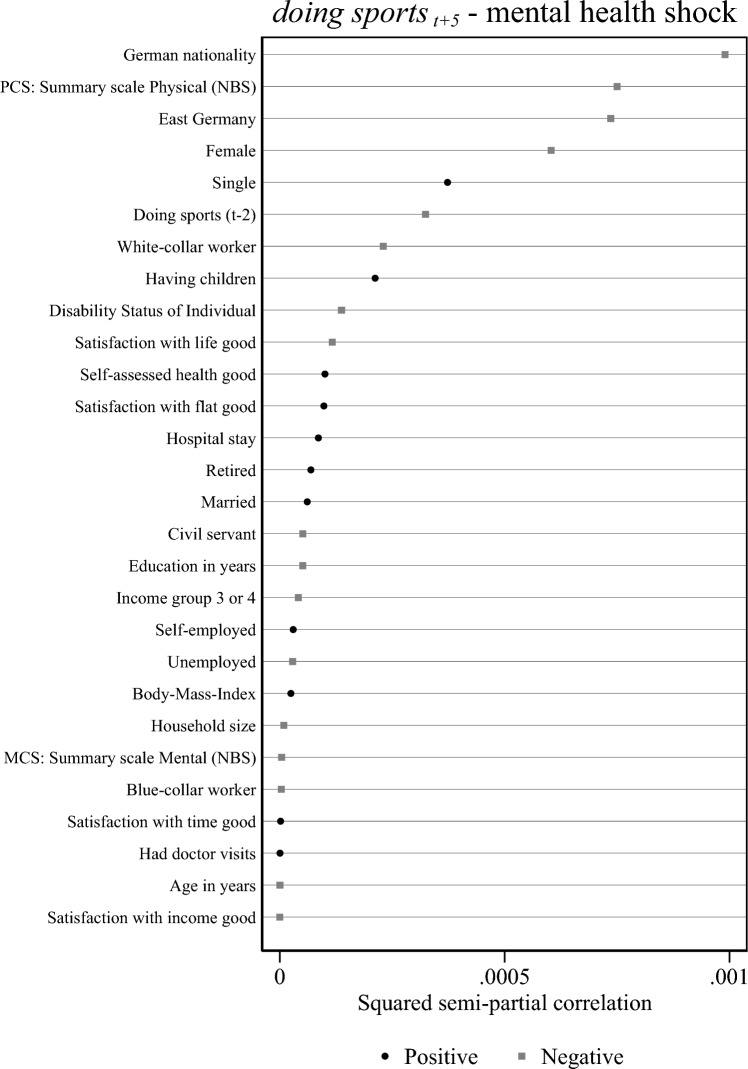
Heterogeneity analysis: Doing sports. Note: while the depicted values represent squared semi-partial correlations, positive and negative distinguishing refers to the sign of semi-partial correlation

Out of the above mentioned dimensions, we focus on gender differences (Fig. 16) and being single (Fig. 17). In addition, we have a closer look at education (Fig. 18), because this possible dimension of effect heterogeneity is of particular importance to Margolis’ [32] line of argument.^32^ The estimation framework we use allows for a straightforward comparison of the effects by discrete variables. Estimating effects at the subgroup level only requires calculating the average (Eq. 1) for sub-samples rather than for the full sample.

All in all, we see little effect heterogeneity in any of the considered dimensions. Finding almost no effects in the pooled sample is hence most likely not due to averaging out strong behavior responses that go into different directions for different exogenously defined subgroups. There are, nevertheless, some interesting phenomena to be described. Women are likely to take up sports after a mental health shock occurred in the mid-term perspective. On the contrary, men are unlikely to do sports at least once a week. Another curious pattern is that not single individuals are significantly more likely to become non-smokers after a shock to physical health, whereas the effect for singles goes into the opposite direction though it is statistically insignificant.

We do not observe significant effect heterogeneity with respect to education unlike Margolis [32]. She finds stronger effects of changes in health capital among individuals with higher levels of education, which is consistent with the line of arguments from Grossman’s model that education enables individuals to better process health-related information, including information linked to a health shock. However, our analysis does not support this finding. While the exact cause of this discrepancy remains unclear, one potential explanation lies in a key difference between our study and Margolis’ (2013), aside from variations in empirical methodology-namely, a divergent definition of a health shock.

## Discussion and limitations

The empirical analysis, taken into account the results of robustness checks, indicates that we see virtually no long-term behavioral response to health shocks. Even if some short-term effects are found, they are quite close to zero and frequently do not survive robustness checks. One may interpret this pattern such that acquiring information—through interactions with medical professionals, for instance—after experiencing a health shock and re-evaluating the consequences of bad habits is not enough for long-term change in behavior. It appears that this, at best, leads to marginal transient behavioral responses. The results from the analysis of effect heterogeneity, however, provide weak indication for social and emotional support improving health behavior. Married individuals tend to be more successful in abstaining from tobacco consumption even several years after a shock to physical health. However, with respect to education, the heterogeneity analysis does not reveal a pattern that would support the argument that more education helps to better interpret the signal associated with a health shock and leads to a stronger behavioral response. All in all, our results are in line with the bulk of the literature that finds that sustained change in health-related behavior is not easily achieved. Even a very salient signal about the vulnerability of one’s own health alone, seems not to be sufficient for accomplishing such change in habits.

**Fig. 28:**
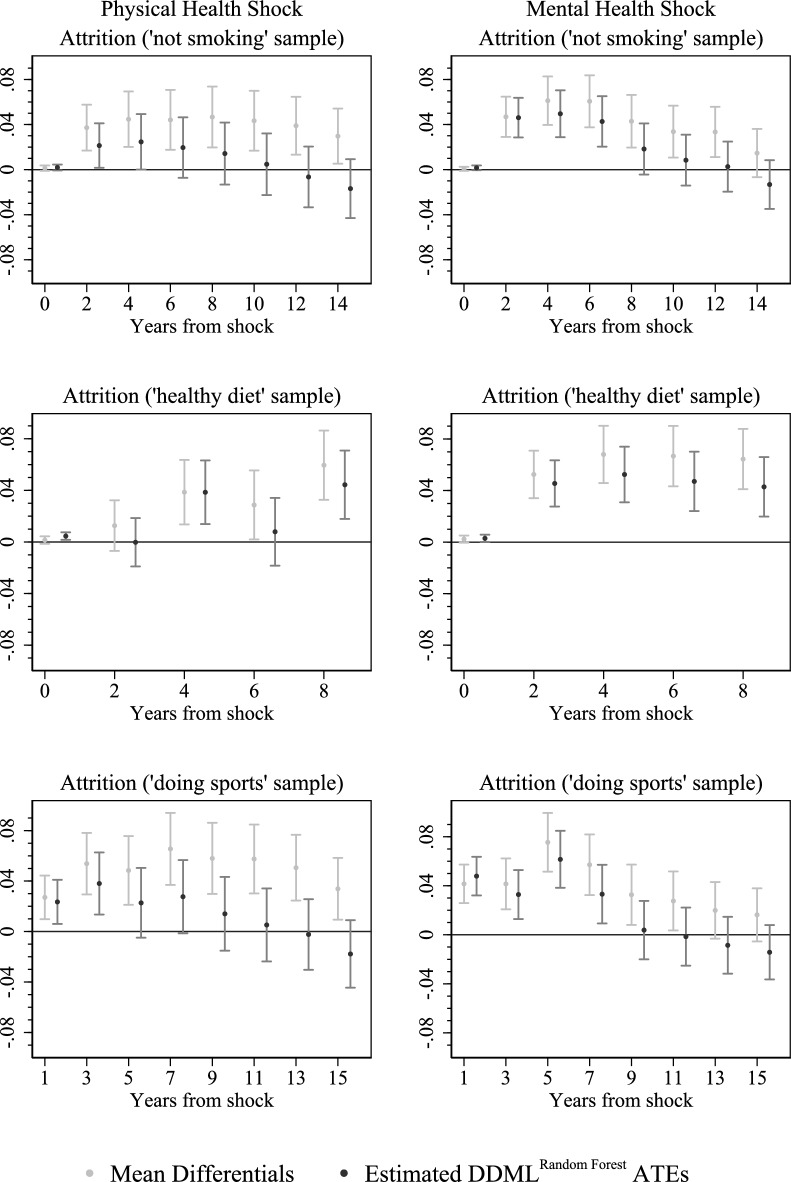
Estimated $\text {DDML}^{\text {RF}}$ ATEs, Endogenous Attrition. Note: 90% confidence intervals are represented by vertical lines

Though our results in qualitative terms prove to be rather robust, the analysis is still subject to some limitations. To begin with, the size of the SOEP is moderate which may make us miss small long-term effects. This is due to panel attrition, which leaves us with relatively small working samples when considering longer time lags between the measurement of the outcome and the health shock. Selective attrition would be an even more fundamental problem. Although we do not see much of a pattern in the data that would argue in this direction, in particular if covariates are taken into account (see Fig. 28), we cannot firmly rule out the possibility that unobservables play a role in attrition, possibly leading to attrition bias. Moreover, the data are self-reported, thus errors coming from misreporting are possible. Furthermore, unlike smoking, the measure of healthy diet is very subjective as health-conscious diet is not clearly defined. Nevertheless, since the analysis focuses on changes in behaviors over time, the subjectiveness of this measure is no obstacle to using it as outcome variable, even if different individuals have different ideas of what a healthy diet is. Only if the occurrence of a health shock changed how health-related behaviors are reported—rather than the behavior itself—this would severely challenge the empirical results. Finally, we do not observe detailed information on the nature of health event and can only infer that an adverse event occurred.^33^

## Conclusion

Using individual level data on health behavior and health indicators from the SOEP during the time span between 2002 and 2019, we assessed the effect of health shocks on lifestyle choices using doubly robust estimating procedure combined with machine learning prediction methods. Although we find some evidence for short-term effects of adverse events, there is however no evidence of persistent effects irrespective of the type of shock or outcome. Though the latter does not prove the absence of such long-term responses we can still rule out strong long-term behavioral changes as a consequence of past health shock. Our results corroborate earlier findings (e.g. [44]), though being somewhat more pessimistic about temporary improvements in health behaviors in response to health shocks. In particular our findings strengthen the earlier result of no maintained behavioral responses, by considering a longer time horizon and a broader spectrum of health behaviors, and by using recently developed methods for estimating the effects of interest. In economic terms this suggest that even a very salient signal of one’s health being vulnerable is not sufficient for initiating such behavioral change. This speaks to the question of whether singular events or transitory interventions are capable of acting as a catalyst for adopting healthy habits. The results of the present analysis cannot support this optimistic view. Moreover, in contrast to previous empirical analyses [32], we find this sobering result similarly for highly and poorly educated individuals. That is, our analysis does not support arguments, in the spirit of Grossman [21], that it is a matter of education whether the health information associated with a health shock is appropriately processed and leads to an adjustment in health behavior.

## Figures and Tables

**Fig. 11:**
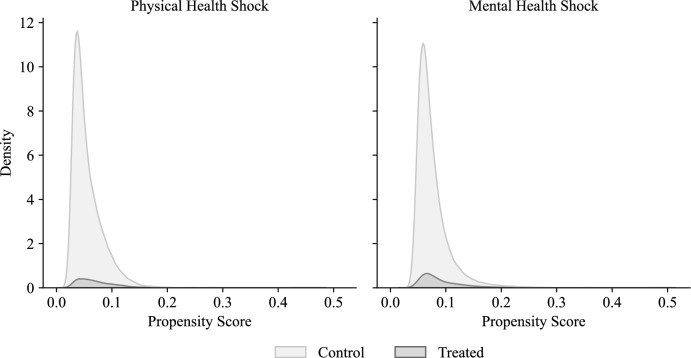
Distribution of propensity score. Note: the distribution is based on all resamples using estimation sample for the outcome *not smoking*_*t*+0_

## Data Availability

This study uses data from the German Socio-Economic Panel (SOEP). SOEP data is not publicly available but can be accessed by researchers upon request to DIW Berlin. Access is provided through a data distribution agreement. Detailed information on data access, conditions of use, and application procedures are available on the SOEP website.

## Appendix A

See Figs. 10, 11, 12, 13, 14, 15, 16, 17, 18, 19, 20, 21, 22, 23, 24, 25, 26, 27, 28 and Tables 2, 3, 4, 5, 6.

**Table 2:** Correlation analysis of shocks

Outcome	Period	N	(1)	(2)	(3)	(4)	(5)	(6)
Not smoking	t	17,356	$-$0.027	$-$0.187	977	1,337	82	0.405
Not smoking	t+2	14,982	$-$0.036	$-$0.194	810	1,097	55	0.550
Not smoking	t+4	12,993	$-$0.046	$-$0.210	691	926	45	0.519
Not smoking	t+6	10,961	$-$0.058	$-$0.209	577	770	40	0.929
Not smoking	t+8	8,873	$-$0.071	$-$0.219	457	631	31	0.779
Not smoking	t+10	7,701	$-$0.075	$-$0.226	394	552	25	0.516
Not smoking	t+12	6,529	$-$0.078	$-$0.226	332	462	21	0.584
Not smoking	t+14	5,437	$-$0.083	$-$0.227	279	401	18	0.544
Healthy diet	t	15,865	$-$0.023	$-$0.160	990	1,333	91	0.355
Healthy diet	t+2	13,567	$-$0.043	$-$0.159	836	1,078	69	0.734
Healthy diet	t+4	11,441	$-$0.062	$-$0.162	679	880	49	0.632
Healthy diet	t+6	9,261	$-$0.066	$-$0.158	552	698	36	0.351
Healthy diet	t+8	8,040	$-$0.066	$-$0.157	447	598	27	0.247
Doing sports	t+1	14,017	$-$0.034	$-$0.193	789	1,057	57	0.729
Doing sports	t+3	12,275	$-$0.044	$-$0.199	666	920	46	0.553
Doing sports	t+5	10,488	$-$0.058	$-$0.211	567	742	36	0.488
Doing sports	t+7	8,884	$-$0.064	$-$0.208	460	636	30	0.586
Doing sports	t+9	7,118	$-$0.066	$-$0.216	364	524	24	0.565
Doing sports	t+11	6,144	$-$0.077	$-$0.224	308	453	20	0.544
Doing sports	t+13	5,147	$-$0.073	$-$0.225	256	383	16	0.456
Doing sports	t+15	4,130	$-$0.078	$-$0.243	211	307	12	0.321

Note: (1)—correlation of PCS and MCS, (2)—correlation of $\frac{\text {PCS}_t - \text {PCS}_{t-2}}{\text {PCS}_{t-2}}$ and $\frac{\text {MCS}_t - \text {MCS}_{t-2}}{\text {MCS}_{t-2}}$, (3)—N with shock to physical health, (4)—N with shock to mental health, (5)—N with shocks to both physical and mental health, (6) - p-value of Chi-squared test

**Table 3:** Count of physical shocks by Count of mental shocks

	**Count of mental shocks**					
**Count of physical shocks**	0	1	2	3	4	Total
0	10,941	3,097	547	61	4	14,650
1	2,186	1,126	284	54	4	3,654
2	310	232	77	11	0	630
3	30	25	20	3	1	79
4	2	2	1	0	0	5
Total	13,469	4,482	929	129	9	19,018

**Table 4:** Sample attrition

Outcome	Shock	Period	$N_{t+s}$	$N_{t+s}/N_{t+0}$	T Y	T N	C Y	C N
Not smoking	Physical	t	17,356	17,356	689	288	11,700	4,679
Not smoking	Physical	t+2	14,982	0.86	0.85	0.78	0.86	0.87
Not smoking	Physical	t+4	12,993	0.75	0.71	0.69	0.75	0.75
Not smoking	Physical	t+6	10,961	0.63	0.60	0.57	0.64	0.63
Not smoking	Physical	t+8	8,873	0.51	0.46	0.47	0.51	0.51
Not smoking	Physical	t+10	7,701	0.44	0.40	0.41	0.45	0.44
Not smoking	Physical	t+12	6,529	0.38	0.34	0.34	0.38	0.37
Not smoking	Physical	t+14	5,437	0.31	0.28	0.29	0.32	0.30
Not smoking	Mental	t	17,356	17,356	896	441	11,493	4,526
Not smoking	Mental	t+2	14,982	0.86	0.82	0.83	0.87	0.86
Not smoking	Mental	t+4	12,993	0.75	0.69	0.70	0.75	0.75
Not smoking	Mental	t+6	10,961	0.63	0.57	0.59	0.64	0.63
Not smoking	Mental	t+8	8,873	0.51	0.46	0.49	0.51	0.51
Not smoking	Mental	t+10	7,701	0.44	0.40	0.44	0.45	0.44
Not smoking	Mental	t+12	6,529	0.38	0.34	0.37	0.38	0.36
Not smoking	Mental	t+14	5,437	0.31	0.29	0.32	0.32	0.30
Healthy diet	Physical	t	15,865	15,865	562	428	7,508	7,367
Healthy diet	Physical	t+2	13,567	0.86	0.84	0.85	0.86	0.85
Healthy diet	Physical	t+4	11,441	0.72	0.68	0.69	0.74	0.70
Healthy diet	Physical	t+6	9,261	0.58	0.55	0.56	0.60	0.57
Healthy diet	Physical	t+8	8,040	0.51	0.45	0.46	0.53	0.49
Healthy diet	Mental	t	15,865	15,865	674	659	7,396	7,136
Healthy diet	Mental	t+2	13,567	0.86	0.81	0.81	0.87	0.85
Healthy diet	Mental	t+4	11,441	0.72	0.66	0.65	0.74	0.71
Healthy diet	Mental	t+6	9,261	0.58	0.51	0.53	0.61	0.57
Healthy diet	Mental	t+8	8,040	0.51	0.44	0.46	0.54	0.49
Doing sports	Physical	t+1	14,017	14,017	150	639	3,901	9,327
Doing sports	Physical	t+3	12,275	0.88	0.88	0.82	0.88	0.85
Doing sports	Physical	t+5	10,488	0.75	0.78	0.69	0.76	0.73
Doing sports	Physical	t+7	8,884	0.63	0.66	0.55	0.67	0.61
Doing sports	Physical	t+9	7,118	0.51	0.57	0.43	0.56	0.48
Doing sports	Physical	t+11	6,144	0.44	0.52	0.35	0.50	0.41
Doing sports	Physical	t+13	5,147	0.37	0.45	0.29	0.43	0.34
Doing sports	Physical	t+15	4,130	0.29	0.35	0.24	0.36	0.27
Doing sports	Mental	t+1	14,017	14,017	293	764	3,758	9,202
Doing sports	Mental	t+3	12,275	0.88	0.86	0.85	0.88	0.85
Doing sports	Mental	t+5	10,488	0.75	0.73	0.67	0.76	0.73
Doing sports	Mental	t+7	8,884	0.63	0.65	0.57	0.67	0.61
Doing sports	Mental	t+9	7,118	0.51	0.56	0.46	0.56	0.48
Doing sports	Mental	t+11	6,144	0.44	0.51	0.39	0.50	0.41
Doing sports	Mental	t+13	5,147	0.37	0.45	0.32	0.43	0.34
Doing sports	Mental	t+15	4,130	0.29	0.38	0.25	0.35	0.27

Note: $N_{t+s}$ is number of observations is $t+s$ period. T stands for treated (had a shock between periods $t+0$ and $t-2$), C—for control (did not have a shock between periods $t+0$ and $t-2$), Y refers to the *’yes’* answer, i.e. having a good habit (not smoking, keeping a health conscious diet, doing sports at least once a week), N—*’no’*. The table reads as follows, e.g. in the sample for analyzing the effect on smoking in $t+0$ period, there were 17,356 individuals in $t+0$ period, out of which 689 had a shock and did not smoke, 288—had a shock and smoked, 11,700—did not have a shock and did not smoke, 4679—did not have a shock and smoke. In the period $t+2$, 86% of the original sample stayed, from the sub sample TY - 85%, TN - 78%, CY - 86%, CN - 87%

**Table 5:** Descriptive statistics of confounders measured pre-shock

Variable	N	mean	sd	min	max
MCS: Summary scale Mental (NBS)	17,356	49.95	10.00	3.53	77.77
PCS: Summary scale Physical (NBS)	17,356	49.82	9.75	9.21	75.46
Body-Mass-Index	17,356	25.62	4.31	11.63	66.02
German nationality	17,356	0.92	0.27	0.00	1.00
East Germany	17,356	0.31	0.46	0.00	1.00
Female	17,356	0.52	0.50	0.00	1.00
Age in years	17,356	48.12	15.64	17.00	97.00
Married	17,356	0.67	0.47	0.00	1.00
Single	17,356	0.18	0.38	0.00	1.00
Having children	17,356	0.35	0.48	0.00	1.00
Household size	17,356	2.77	1.27	1.00	12.00
Education in years	17,356	12.18	2.64	7.00	18.00
Self-employed	17,356	0.06	0.25	0.00	1.00
Blue-collar worker	17,356	0.17	0.37	0.00	1.00
White-collar worker	17,356	0.30	0.46	0.00	1.00
Civil servant	17,356	0.05	0.22	0.00	1.00
Unemployed	17,356	0.06	0.25	0.00	1.00
Retired	17,356	0.17	0.37	0.00	1.00
Hospital stay	17,356	0.12	0.32	0.00	1.00
Disability Status of Individual	17,356	0.10	0.30	0.00	1.00
Self-assessed health good	17,356	0.50	0.50	0.00	1.00
Self-assessed health satisfactory	17,356	0.34	0.47	0.00	1.00
Self-assessed health poor	17,356	0.15	0.36	0.00	1.00
Satisfaction with life good	17,356	0.04	0.20	0.00	1.00
Satisfaction with life satisfactory	17,356	0.26	0.44	0.00	1.00
Satisfaction with life poor	17,356	0.70	0.46	0.00	1.00
Satisfaction with income good	17,356	0.10	0.30	0.00	1.00
Satisfaction with income satisfactory	17,356	0.33	0.47	0.00	1.00
Satisfaction with income poor	17,356	0.57	0.50	0.00	1.00
Satisfaction with flat good	17,356	0.03	0.18	0.00	1.00
Satisfaction with flat satisfactory	17,356	0.16	0.37	0.00	1.00
Satisfaction with flat poor	17,356	0.81	0.40	0.00	1.00
Satisfaction with time good	17,356	0.08	0.28	0.00	1.00
Satisfaction with time satisfactory	17,356	0.27	0.44	0.00	1.00
Satisfaction with time poor	17,356	0.65	0.48	0.00	1.00
Had doctor visits	17,356	0.68	0.47	0.00	1.00
Income group 1	17,356	0.23	0.42	0.00	1.00
Income group 2	17,356	0.23	0.42	0.00	1.00
Income group 3	17,356	0.25	0.44	0.00	1.00
Income group 4	17,356	0.28	0.45	0.00	1.00

Note: the results are based on the sample for analyzing the effect on smoking in $t+0$ period

**Table 6:** Sample size for alternative definitions of a shock

Threshold	$N^{PCS}_{treated}/ N$	$N^{MCS}_{treated}/ N$
$-$0.01	0.46	0.48
$-$0.02	0.43	0.45
$-$0.03	0.41	0.42
$-$0.04	0.39	0.38
$-$0.05	0.36	0.35
$-$0.06	0.34	0.32
$-$0.07	0.32	0.30
$-$0.08	0.30	0.27
$-$0.09	0.27	0.25
$-$0.10	0.26	0.22
$-$0.11	0.24	0.20
$-$0.12	0.22	0.19
$-$0.13	0.20	0.17
$-$0.14	0.19	0.15
$-$0.15	0.18	0.14
$-$0.16	0.16	0.13
$-$0.17	0.15	0.12
$-$0.18	0.14	0.11
$-$0.19	0.13	0.10
$-$0.20	0.12	0.09
$-$0.21	0.11	0.08
$-$0.22	0.10	0.07
$-$0.23	0.09	0.07
$-$0.24	0.08	0.06
$-$0.25	0.08	0.05
$-$0.26	0.07	0.05
$-$0.27	0.07	0.04
$-$0.28	0.06	0.04
$-$0.29	0.06	0.04
$-$0.30	0.05	0.03
$-$0.31	0.05	0.03
$-$0.32	0.04	0.03
$-$0.33	0.04	0.02
$-$0.34	0.04	0.02
$-$0.35	0.03	0.02
$-$0.36	0.03	0.02
$-$0.37	0.03	0.02
$-$0.38	0.03	0.01
$-$0.39	0.02	0.01
$-$0.40	0.02	0.01

Note: the results are based on the sample for analyzing the effect on smoking in $t+0$ period

## Footnotes

1 In her empirical analysis, which unlike ours is concerned with more specific health events namely new chronic conditions, Margolis [32] indeed finds heterogeneity with respect to education. In contrast, the present analysis—using very different empirical methods—finds little effect heterogeneity, with education not being among the most important heterogeneity-dimensions; see Sect. 4.3.

2 See also Sundmacher [44], who links her empirical analysis also to these strands of economic theory.

3 Even accidents ([17], cf.) cannot be regarded as such source of exogenous variation, because they are likely to impact on various outcomes through other channels rather than only health.

4 This time frame is determined by the availability of the variables (introduced to SOEP in 2002) that are used to construct the key explanatory variables. Additionally, we use data from 2001 to condition on pre-shock values of the outcome variable for one of the healthy behaviors (explained in more detail later).

5 We think of short-term effect as within two years or less, mid-term as within 3–6 years, and long-term as within more than 6 years. We do not condition on health shocks that may hit an individual in later years, i.e. we do not want to compare individuals hit by a shock to individuals never hit, but examine how the effect of shock at a particular point in time evolves over time.

6 Note that if the outcome variable is missing in, e.g. $t+4$, it might still be observed for the same individual in, e.g. $t+6$.

7 Even though it is a bit counterintuitive to use negation, namely "not smoking", instead of "smoking", we prefer it in order to unify the direction of the effects, i.e. the larger the better.

8 The question is asked irregularly, namely, in 2001, 2003, 2005, 2007, 2008, 2009, 2011, 2013, 2015, 2017, 2018, 2019, yet is present in more odd years than in even.

9 The samples for estimation the effect of mental and physical shock are equivalent in this regard, therefore we only provide visualization for a shock to physical health.

10 The share of smoking individuals measured pre-shock is 30%, keeping a health-conscious diet is 51%, doing sports at least once a week is 26% (based on the samples for analyzing instantaneous effects).

11 The reason for—depending on the considered outcome—looking on different periods in which a health shock may have occurred is that we need to control for the lifestyle behavior prior to a possible shock. Yet, unlike physical exercise and smoking, information on dietary preferences not available for year before 2004.

12 The results are based on the samples for analyzing the effect of a shock on smoking behavior.

13 Prior selection of potential controls is not uncommon in application of machine learning algorithms to survey data, (e.g. [2, 6, 22, 26]).

14 That is, we use the largest estimation sample for the outcome *not smoking.*

15 $\text {ASMD} = {\left| \bar{x}_{\text {treat}, m} - \bar{x}_{\text {control},m}\right| }/{\sqrt{0.5 s^2_{\text {treat}, m}+0.5 s^2_{\text {control}, m}}}$, where *m* indexes the covariates, $\bar{x}$ is the sample mean, $s^2$ is the sample variance and $\textit{treat}$ and $\textit{control}$ indicate the sub-﻿samples of individuals hit and not hit by the shock.

16 Fig. 5 depicts *absolute* mean differences and is, hence, not informative about the sign of correlation between education and treatment status. Yet, regressing the health shock indicators on years of education yields negative and statistically significant coefficients.

17 Evidently, condition on observables does not eliminate possible selection bias that operates through unobservables. Nonetheless, making use of numerous possible conditioning variables in a data-driven way shrinks the margin for selection on unobservables generating a major bias.

18 Corresponding results for the ATT (average treatment effect on the treated) are available upon request. These estimates differ surprisingly little from their ATE counterparts.

19 SUTVA appears to be least critical in the present application. Health shocks are personalized events, and spillovers from health shocks others experience may—if at all—occur within a household but not across the SOEP households. The common support assumption can be checked by examining the distribution of the estimated propensity scores. Yet, since we use machine learning methods that rest on numerous sample splits, the distributions found in some of these samples will almost certainly argue against common support. The focus of our approach is on fostering confidence in CIA, by combining a doubly robust estimator with machine learning.

20 The AIPW shares the double robustness property with, for instance, regression adjusted inverse probability weighting estimation.

21 We choose random forest due to its ability to capture complex, non-linear relationships.

22 LASSO was chosen because of its strength in high-dimensional variable selection.

23 The distributions of propensity score for the biggest estimation sample (outcome $not \, smoking_{t+0}$) are presented in Fig. 11.

24 E.g. latent health deficits may eventually result in a physical health shock and also deter individuals from physical exercise.

25 Still, it needs to be stressed that a health conscious diet and weight loss are different outcomes, which do not one-to-one correspond. Furthermore the analysis of Sundmacher [44] differs from ours in various respects, e.g. she considers the years 1999-2008, and uses a one point reduction in self-assessed health as measure of an adverse health event.

26 The penalty term is chosen from 10 numbers spaced evenly on a log scale between 0.001 and 1 using 5-folds cross validation.

27 As the estimation with extensive hyperparameter tuning is computationally expensive, we reduce the grid of the maximum depth to only two values, namely 4 and 6, and reduced the number of cross-validation folds to 2.

28 For the immediate ($t=0$) effects on attrition in the samples used to analyze *not smoking* and *healthy diet*, Fig. 28 shows precisely estimated zeros. This pattern occurs for purely technical reasons: we only observe a health shock (or its absence) if an individual enters the survey at $t=0$. Consequently, there is no attrition in the strict sense at $t=0$, and missing information on the respective outcome only results from item non-response, which is not too common in the data.

29 The individual effects are averaged over resamples.

30 Formally, $SPC_j^2 = \frac{t_j^2(1-R^2)}{N-k}$ where $R^2$ is the R squared of the model with *k* variables, $j = 1,..., k$ with *k* being the number of covariates, *t* is t-statistic of the *j*’s variable, and *N* is the number of observations [43].

31 Namely, $not \, smoking_{t-4}$— physical health shock, $not \, smoking_{t-4}$—mental health shock, $healthy \, diet_{t-2}$ – physical health shock, $healthy diet_{t-4}$ – mental health shock, $doing \, sports_{t-5}$—physical health shock, $doing \, sports_{t-5}$—mental health shock.

32 In order to perform group analysis, we divided the sample into those who have 12 years of education or more and those who have less. The threshold of 12 is based on the number of years needed to obtain Abitur.

33 However, the data show a statistically highly significant correlation between health shocks, as defined in this analysis, and both the length and frequency of hospital stays.
